# Particle-associated diazotrophs drive nitrogen fixation in Arctic subsurface waters of the Barents Sea

**DOI:** 10.1038/s41467-026-75354-5

**Published:** 2026-07-08

**Authors:** Mar Benavides, Arthur Coët, Marta Sebastián, Angela Vogts, Christian Furbo Reeder, Elena Cerdán-García, Louisa Norman, Ruth Hawley, Oliver Flanagan, Maeve C. Lohan, Joanne E. Hopkins, Claire Mahaffey

**Affiliations:** 1 National Oceanography Centre, European Way, Southampton, SO14 3ZH UK; 2 Aix Marseille Univ, Université de Toulon, CNRS, IRD, MIO, Marseille, France; 3 Aix Marseille Univ, CNRS, CINAM, Turing Centre for Living Systems, Marseille, France; 4 Departament de Biologia Marina i Oceanografia, Institut de Ciències del Mar, Consejo Superior de Investigaciones Científicas (CSIC), Barcelona, Spain; 5 Department of Biological Oceanography, Leibniz Institute for Baltic Sea Research, Rostock, Germany; 6 Department of Biology and Environmental Science, Center for Ecology and Evolution in Microbial Model Systems - EEMIS, Linnæus University, Kalmar, Sweden; 7 Department of Earth, Ocean and Ecological Sciences, 4 Brownlow Street, University of Liverpool, Liverpool, L69 3GP UK; 8 School of Ocean and Earth Science, University of Southampton, Southampton, SO14 3ZH UK

**Keywords:** Marine biology, Water microbiology

## Abstract

Biological dinitrogen (N_2_) fixation sustains productivity in oligotrophic oceans and is now also thought to contribute substantially to the nitrogen supply in the warming Arctic. Here we demonstrate significant N_2_ fixation by particle-associated diazotrophs in subsurface waters of the Barents Sea. Comparing our findings with subtropical studies reveals particle-associated non-cyanobacterial diazotrophs as the primary N_2_ fixers in subsurface Arctic waters of the Barents Sea, contrasting with diverse communities in warmer regions. As the Arctic shifts towards oligotrophication, understanding the magnitude and controls of particle-associated N_2_ fixation will provide critical insights into future nitrogen supply required to sustain productivity in the rapidly changing Arctic Ocean. However, particle-associated N_2_ fixation may be a distinctive feature of the Barents Sea, where in contrast to other Arctic shelves the seasonal and long-term trends in nitrogen dynamics are heterogeneously determined by changes in the external Atlantic Water supply, sea-ice extent, and terrestrial inputs. In this context, the role of particle-associated N_2_ fixation across the wider Arctic Ocean will require further investigation.

## Abstract (web summary)

The study shows that particle-associated non-cyanobacterial diazotrophs dominate nitrogen fixation in subsurface Barents Sea waters, contrasting with warmer oceans and highlighting an important Arctic source of new nitrogen for productivity.

## Introduction

Dinitrogen (N_2_) fixation by diazotrophic microorganisms constitutes the main source of reactive nitrogen in the ocean, where it sustains primary productivity and carbon export^1,2^. Traditionally, N_2_ fixation was considered largely restricted to oligotrophic, warm tropical and subtropical waters where reactive nitrogen limitation favours diazotroph activity. However, several studies have expanded this paradigm, documenting significant N_2_ fixation rates at higher latitudes, including temperate and polar regions^1^. The Arctic has warmed three to four times faster than the global average since 1979 due to Arctic ‘amplification’ (ice albedo feedback and heat transport^3,4^) and ‘atlantification’ (warm Atlantic origin water spreading further into the Arctic basin subsurface^5^). The Barents Sea, the shelf sea gateway through which the most important imports of Atlantic-origin nitrate into the Arctic take place^6^, is experiencing the most rapid warming and sea-ice loss. Continued warming leads to glacier and sea-ice melting, which releases nutrients and iron^7^ advancing the onset and increasing the magnitude of phytoplankton blooms^8,9^. The extent of ice-free waters increases over the year, enhancing nitrogen depletion by phytoplankton and eventually leading to decreased primary productivity and carbon export^10,11^.

The progressively earlier seasonal depletion of nitrogen in the Arctic may expand the niche for diazotrophs^12,13^. A preliminary study estimated Arctic N_2_ fixation supplies 9.2 Tg N y^−1^ globally representing 7.1% of the global input^14^, and current ecosystem models predict an increase of diazotrophs in Arctic latitudes towards the end of the 21^st^ century^15^. More recent studies have documented active N_2_ fixation across diverse Arctic Ocean regions, including the Chukchi Sea, Central Arctic, Eurasian marginal ice zones, and Fram Strait^16–19^. The only specific N_2_ fixation measurements available in the region show that the nitroplast (formerly UCYN-A) of the eukaryotic alga *Braarudosphaera bigelowii*^20^ actively fixes N_2_ in the Pacific sector of the Arctic at rates similar to those of (sub)tropical waters^19^. Despite the cosmopolitan distribution of this nitroplast across latitudes globally^21^, non-cyanobacterial diazotrophs (NCDs) overwhelmingly dominate *nifH* gene sequences and expression in the Arctic, particularly in its Atlantic sector^16,17,22–25^ but also in some parts of the Chukchi and Beaufort Seas^26^. Contrary to diazotrophic cyanobacteria, NCDs do not have photosynthetic machinery^27^ and based on their genomes, are predicted to rely on other external sources of carbon and energy^28^. Organic particles are rich in carbon and can harbour low oxygen microzones, making them ideal niches for NCDs to fix N_2_^29,30^. Recent studies have demonstrated that gammaproteobacterial NCDs fix N_2_ in association with particles in the subtropical North Pacific^31,32^, but the recurrence and N_2_ fixation potential of these associations in the Arctic is unknown.

Arctic waters with seasonal ice coverage export almost twice as many particles as ice-free zones^33^, and combined with the Arctic’s extremely broad shelf areas, supply coastal waters with substantial volumes of sediment-laden particles rich in potentially bioavailable iron and carbon^34^ - key resources enabling NCDs to thrive and perform N_2_ fixation^28,29^. Here, we aimed to evaluate the N_2_ fixation potential and the identity of particle-associated NCDs in the Barents Sea, a region of the Arctic characterised by rapid warming, sea-ice loss, and high primary productivity^9^. Despite efficient nitrogen recycling that can replenish seasonal nitrate uptake needs^35^, the system has experienced decadal-scale nitrate depletion throughout the water column^36^. Combining single-cell spectrometry, immunofluorescence and molecular methods, we find that particle-associated N_2_ fixation in the subsurface waters of the Barents Sea is predominantly driven by NCDs. By comparing our findings with subtropical studies, we demonstrate that particle-associated NCDs stand out as the dominant diazotrophs in the Arctic’s Barents Sea subsurface waters, unlike the more diverse diazotroph communities reported from warmer regions. As a region at the forefront of Arctic oligotrophication^37,38^, the magnitude and controls of particle-associated N_2_ fixation in the Barents Sea provide hints on the future of reactive nitrogen supply in this globally climate-regulating region.

## Results and discussion

**Fig. 1:**
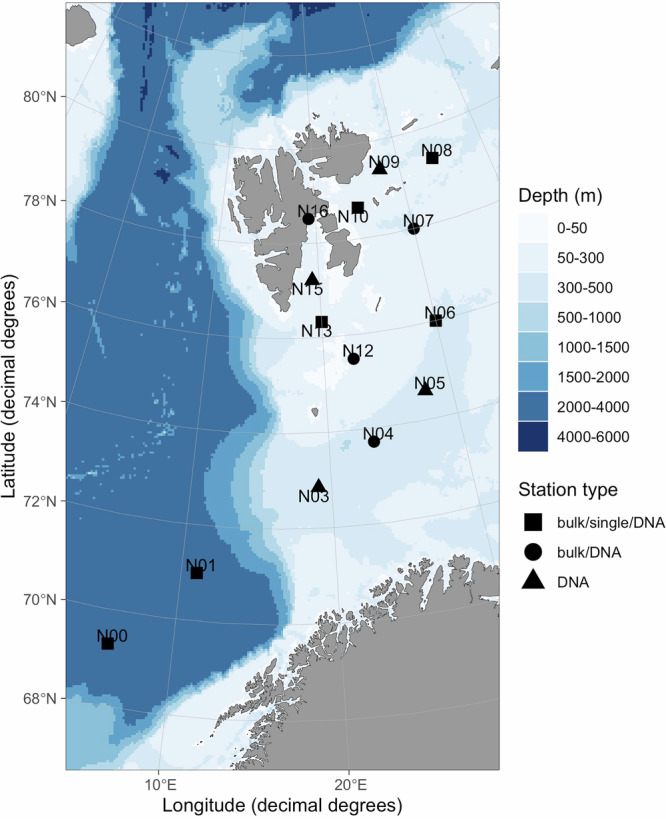
Sampling campaign map. Map showing stations sampled for bulk N_2_ fixation rates, single-cell N_2_ fixation rates, and DNA (bulk/single/DNA), bulk N_2_ fixation rates and DNA (bulk/DNA), or only DNA, during the DY167 N-ARC cruise.

We sampled at fourteen stations aiming at covering the Atlantic and Arctic influenced end-members of the Barents Sea^39,40^, including Atlantic Water, warm Atlantic Water and Arctic Water masses (Supplementary Results, Fig. 1, Supplementary Data 1). Suspended, slow and fast sinking particles were collected using a marine snow catcher (MSC) deployed 10 m below the deep chlorophyll maximum^41^, ranging from 22 to 66 m during our cruise (Supplementary Data 1; Fig. S1).

Bulk measurements across individual MSC fractions revealed that the suspended fraction had the highest N_2_ fixation rates (up to 1.62 nmol N l^−1^ d^−1^), followed by the fast sinking fraction (up to 0.46 nmol N l^−1^ d^−1^), and the slow sinking fraction (up to 0.22 nmol N l^−1^ d^−1^) (Fig. S2, Supplementary Data 2). These rates are five to ten times lower than previous measurements in surface waters of the Bering, Chukchi, and Beaufort Seas (compiled in ref. ^12^), but in the range of surface water bulk N_2_ fixation rates during our cruise in the Barents Sea (Mahaffey et al., in prep.). Based on the bulk N_2_ fixation rates observed, a subset of five stations (N00, N06, N08, N10, and N13; Fig. 1, Supplementary Data 1) were selected for single-cell analysis. Subsamples from each MSC fraction were size-fractionated to discriminate particle-associated ( > 3 µm) from free-living cells ( < 3 µm and >0.2 µm) in NifH antibody staining and *nifH* amplicon sequencing analyses. Because our focus was to measure particle-associated N_2_ fixation, only 3 µm filters were used for single-cell N_2_ fixation analyses (see Methods and Supplementary Information).

**Fig. 2:**
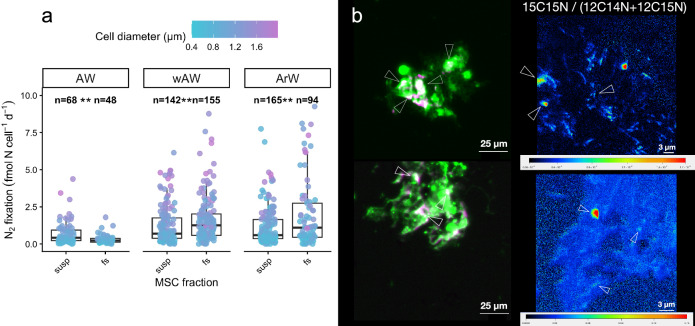
Particle-associated N_2_ fixation rates in subsurface waters. **a** Particle-associated single-cell N_2_ fixation rates (boxplots) overlaid by cell diameter (circles) in the suspended (susp) and fast sinking (fs) marine snow catcher particle fractions, faceted by water mass (AW = Atlantic Water, wAW = warm Atlantic Water, ArW = Arctic Water). In the boxplots, the centre line indicates the median, the box spans the interquartile range (25th–75th percentiles), and the whiskers extend to the most extreme observations within 1.5 × IQR of the quartiles. All observations are overlaid as jittered points. Significant differences calculated by Kruskal-Wallis tests are depicted with asterisks (**p < 0.01; AW *p* = 0.00066, wAW *p* = 0.00164, ArW *p* = 0.00441). The total number of cells analysed was 729 (Supplementary Data 3) but only rates up to 10 fmol N cell^-1^ d^−1^ are shown in this plot. Only cells with diameters <2 µm, representing 85% of the cells analysed (*n* = 672), are shown in this figure. **b** examples of immunofluorescence microscopy images showing NifH positive cells in pink and DAPI positive cells in green (left) mapped to ^15^N-enriched cells analysed by nanoSIMS (right, pointed by arrows) mapped to (right). White cells in immunofluorescence images are the overlap of both NifH and DAPI-positive cells (left column of (**b**)). Cells actively fixing N_2_ are pointed by arrows in nanoSIMS images (right column of (**b**)). All the data shown in this figure were obtained from 3 µm filters, considered as particle-associated cells.

Measuring N_2_ fixation rates of individual NCDs taxa usually requires combining oligonucleotide probing (catalysed reporter deposition-fluorescence in situ hybridisation or CARD-FISH) with nanoscale secondary ion mass spectrometry (nanoSIMS; e.g., refs. ^31,32^). While this approach resolves relatively broad taxonomic groups, it does not specifically capture the broader NCDs assemblage, which spans multiple taxonomic lineages and is widespread across the global oceans^28^. Here, we applied a nitrogenase iron protein (NifH) antibody staining approach able to target virtually all diazotrophs independently of their taxonomy^42^. Based on NifH immunofluorescence counts on 3 and 0.2 µm filters, we found that particle-associated diazotrophs (3 µm filters) comprised 0.25–9.26% of the total microbial community (i.e., 0.2 and 3 µm filters together), with the highest proportions observed at stations in warm Atlantic Water (Fig. S2), situated near the Polar Front^43^. NifH-immunofluorescence-positive cells in the slow sinking fraction were exclusively detected in warm Atlantic Water waters (Fig. S3), corresponding to the lowest bulk N_2_ fixation rates (Fig. S2); consequently, this fraction was excluded from subsequent nanoSIMS analyses. Mapping immunolabeled cells in 3 µm filters for nanoSIMS, we measured particle-associated N_2_ fixation rates on 729 individual cells. Most of the rates ranged between 0 and 10 fmol N cell^−1^ d^−1^, although a peak value of 402 fmol N cell^−1^ d^−1^ was observed in the fast sinking fraction of station N10 corresponding to a cell measuring 5.24 µm in diameter (Supplementary Data 3). We note that the single-cell N_2_ fixation rates reported here are conservative since immunolabeling may have diluted the ^15^N signal of cells by ~15%, consistent with previous CARD-FISH applications^44^ (see Supplementary Information). Averaging particle-associated N_2_ fixation rates per water mass showed an increase from Atlantic Water (0.56 ± 0.67 fmol N cell^−1^ d^−1^, *n* = 130), through warm Atlantic Water (1.95 ± 5.67 fmol N cell^−1^ d^−1^, *n* = 319), to Arctic Water in the inner Barents Sea (4.53 ± 14.10 fmol N cell^−1^ d^−1^, *n* = 280), being significantly higher in fast sinking than in suspended particles (Kruskal-Wallis test, **p < 0.01) (Fig. 2). These rates are higher than those measured on two different *B. bigelowii* nitroplast lineages in the Bering and Chukchi Seas (7.6 and 13.0 fmol N cell^-1^ d^-1^, respectively^19^), but similar to NCDs’ single-cell N_2_ fixation rates measured in the North Pacific Ocean (67−121 fmol N cell^−1^ d^−1^.^32^). This suggests that particle-associated NCDs exhibit consistent N_2_ fixation performance across latitudes. Future assessments of the contribution of particle-associated activity to overall N_2_ fixation will require detailed knowledge of particle size spectra and abundance in the water column, obtained using optical methods such as Laser In-Situ Scattering and Transmissometry^32^.

Although the immunolabeling approach used here detects virtually all diazotrophs synthesising the NifH protein, it cannot distinguish between taxa, complicating the determination of whether particle-associated diazotrophs were exclusively NCDs or also included diazotrophic cyanobacteria, as the latter are often observed embedded in particles in low latitude waters^2,45,46^. In this case, cell size can be helpful to discriminate cyanobacteria from NCDs. Although we measured a few outlier cells with diameters of up to 5.24 µm (Supplementary Data 3), 85% of the cells analysed had diameters ranging from 0.4 to 2 µm (Fig. S4). Cells within this size range could potentially correspond to the *B. bigelowii* nitroplast, which is known to fix N_2_ in the Arctic^19^ and was also identified in surface waters during our cruise (Mahaffey et al., in prep.). However, we can confidently exclude the nitroplast as a contributor to N_2_ fixation in subsurface waters as no corresponding *nifH* gene sequences were detected in our MSC samples (Supplementary Data 4). The sporadic higher rates measured in cells >2 µm could be attributed to the unicellular cyanobacterium *Cyanothece* (Amplicon Sequence Variant (ASV)51, Supplementary Data 3), which has been previously observed in Arctic seawater and sea ice brine^47^. Given that most of the active N_2_-fixing cells detected in our study were <2 µm (Fig. S4) and diazotrophic cyanobacteria (typically >4 µm) were only sparsely detected in *nifH* gene sequences (Supplementary Data 4), we can confidently conclude that particle-associated N_2_ fixation in our study was chiefly driven by NCDs.

**Fig. 3:**
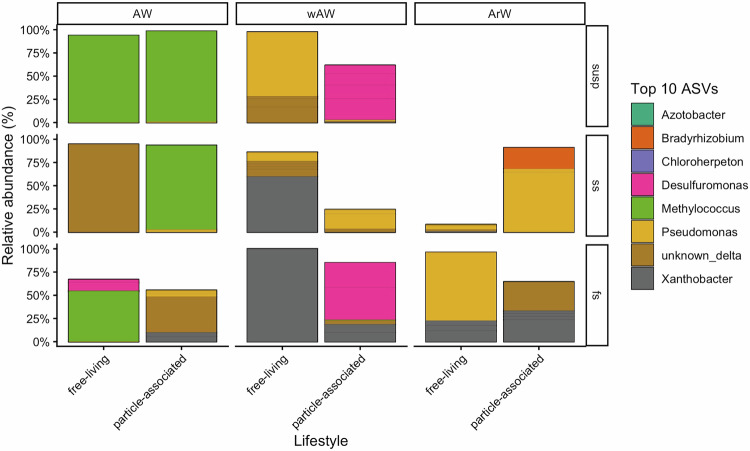
Free-living and particle-associated diazotroph community composition in subsurface waters. Free-living (0.2 µm filters) and particle-associated (3 µm filters) *nifH* gene relative abundance of the top 10 amplicon sequence variants (ASVs) annotated at the class and genus levels, faceted by water mass (AW Atlantic Water, wAW warm Atlantic Water, ArW Arctic Water; see Fig. 1). The full list of annotated ASVs is displayed on Supplementary Data 4.

The top ten ASVs belonged to putative motile genera (Fig. 3), which would support a particle-associated lifestyle^48^. *Azotobacter* was mostly detected in the free-living fraction (i.e., 0.2 µm filters) across all water masses sampled (Fig. 3). This genus has been previously reported from Arctic melt ponds, water under ice, and algal aggregates^24^, and is able to use carbohydrates and organic acids as sources of carbon. *Desulfuromonas*, which are sulphate-reducers and typical of oxygen deficient waters^49^, were particularly abundant in particle-associated fractions of warm Atlantic Water waters where the MSC deployment depth (i.e., 10 m below the deep chlorophyll maximum) coincided roughly with the oxycline (Figs. S1, S5). *Bradyrhizobium* was more evenly detected across water masses, while *Methylococcus* was clearly prevalent in Atlantic Water (Fig. 3, Fig. S5). Active N_2_-fixing Bradyrhizobia species have been found in driftwood around Svalbard^50^, while *Methylococcus* are typically found in sediment and wetland ecosystems^51,52^. *Xanthobacter*, a soil methylmercury degrading bacterium found even in deep bathypelagic waters of the Arctic^53^, was predominant in warm Atlantic Water (Fig. 3) were sedimentary iron and manganese inputs were observed^54^. This suggests that the samples collected included particles of terrestrial and/or glacial origin, supporting the increasingly recognised role of these materials as a source of diazotrophs in pelagic Arctic waters^22,26^. It is also consistent with the well-established role of sea ice and ice-rafted sediments as vectors for the transport of microbial communities from terrestrial and cryospheric environments into open Arctic Ocean^55^.

Particles in the Arctic Ocean originate from diverse sources, including phytoplankton, macroalgae, and glacial sediment runoff^56–58^. However, phytoplankton derived particulate organic carbon export is usually minimum in the boreal summer^59^, when our cruise took place. Over the summer months, as Arctic phytoplankton blooms decline and are exported from surface waters, particles of terrestrial and/or glacial origin likely dominate the water column, as suggested by the diazotroph community composition in our samples (Fig. 3), which resembles sediment-associated assemblages. Alternatively, the scarcity of typical particle-associated pelagic diazotroph communities in our data may reflect the absence of phytoplankton-derived particles at our sampling depth (10 m below the deep chlorophyll maximum, 22-66 m; Supplementary Data 1, Fig. S1) due to remineralisation, consistent with particulate organic carbon attenuation horizons as shallow as 30-60 m in the Barents Sea^60^.

**Fig. 4:**
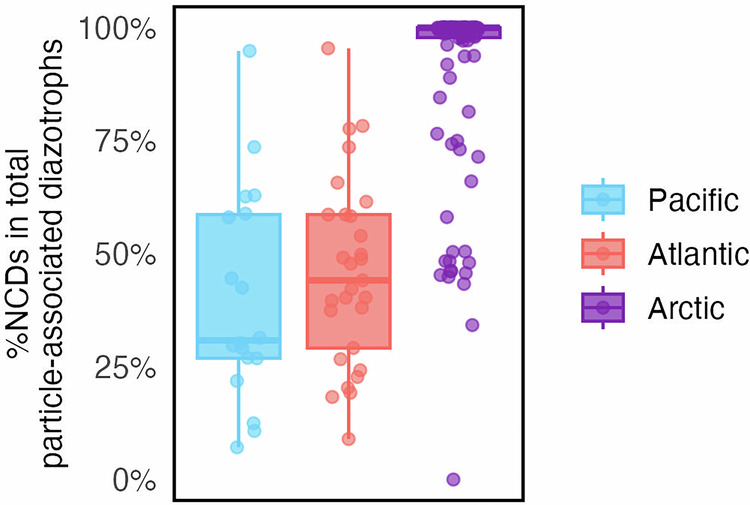
Comparison of particle-associated diazotrophs in subsurface waters across ocean basins. Contribution of particle-associated NCD *nifH* genes to the total particle-associated diazotroph *nifH* gene pool in MSC deployments in the subtropical North Pacific^32^ (*n* = 18) and North Atlantic waters (*n* = 29)^61^, as compared to this study (Arctic, *n* = 129). In the boxplots, the centre line indicates the median, the box spans the interquartile range (25th–75th percentiles), and the whiskers extend to the most extreme observations within 1.5 × IQR of the quartiles. All observations are overlaid as jittered points.

Comparing this study with studies in the North Pacific^32^ and North Atlantic^61^ (which employed the same MSC sampling strategy at 10 m below DCM; see Supplementary Information) reveals that particle-associated diazotroph communities in our Arctic study are remarkably different from those of subtropical regions (Fig. S6A), showcasing a high degree of endemicity (Fig. S6B). We find that while gamma- and betaproteobacteria were present in particle-associated diazotroph communities across the North Pacific, North Atlantic, and Arctic sites, alphaproteobacterial and Desulfuromonadia were not detected in the North Pacific (Fig. S6C). Interestingly, cyanobacteria dominated the particle-associated diazotroph community in subsurface waters of both the North Pacific and North Atlantic, whereas they were virtually absent in the Arctic, where NCDs comprised nearly 100% of the particle-associated diazotroph community (Fig. 4). This aligns with recent modelling efforts showing that particle-associated NCDs are best suited for fixing N_2_ at low temperatures as compared to cyanobacterial diazotrophs^62^.

The Barents Sea is the fastest-warming region of the Arctic and is becoming increasingly nitrogen-limited^36,63,64^, potentially enhancing conditions favourable for N_2_ fixation. The anticipated transition to an increasingly ice-free Barents Sea is expected to further extend this oligotrophic summer period, characterised by low algal biomass and reduced vertical particulate organic carbon flux^10^. These conditions suggest that the potential for N_2_ fixation in the Barents Sea may differ from that in other Arctic regions, with particle-associated NCDs potentially linked to terrestrial and glacial runoff emerging as important contributors to nitrogen availability.

## Methods

### Particle sampling

The DY167 N-ARC GEOTRACES GApr19 cruise took place from 9 July to 13 August 2023 onboard the RRS *Discovery*. A marine snow catcher (MSC; OSIL, Havant, Hampshire, UK) was deployed at 10 m below the deep chlorophyll maximum at fourteen stations (Fig. 1, Supplementary Data 1). After 2 h of settling on deck, the suspended, slow sinking and fast sinking particle fractions were subsequently sampled. The suspended fraction was sampled in nine 4000 ml polycarbonate bottles, where three bottles were used for N_2_ fixation rate measurements (see below), three bottles for DNA sampling, and three bottles for particulate matter natural ^15^N atom % enrichment measurements. The slow sinking and fast sinking fractions were sampled in triplicate 500 ml polycarbonate bottles and 40 ml glass flasks, respectively, for N_2_ fixation measurements. The content of the other triplicate sets of 500 ml polycarbonate bottles and 100 ml glass flasks was size-fractionated by filtration through 3 and 0.2 µm filters and used for DNA extraction and *nifH* gene amplification (Supplementary Information).

### Bulk N_2_ fixation rates

Each MSC fraction was incubated in triplicate with 10% v/v ^15^N_2_-enriched filtered seawater for 48 h in a controlled temperature room adjusted to the temperature of each sampling depth at each station (Supplementary Data 1). Enriched seawater was prepared from 2300 ml seawater, filtered through a 0.2 µm pore size Sartobran PTFE cartridge (Sartorius, Göttingen, Germany), injected with 25 ml 98 atom % ^15^N_2_ gas (Cambridge Isotope Laboratories, Tewksbury, MA, USA), and stirred on a magnet stirred plate overnight. After incubation, 4000 ml, 500 ml, and 30 ml from the suspended, slow sinking and fast sinking fractions, respectively, were filtered onto pre-combusted (450 °C, 6 h) GFF filters (Whatman, Little Chalfont, Buckinghamshire, UK) and stored at -20 °C. Filters were dried at 60 °C for 24 h, pelletised and analysed on an INTEGRA2 Sercon elemental analyser coupled to an isotope ratio mass spectrometer (Crewe, Cheshire, UK) for particulate ^15^N/^14^N isotope ratios, particulate carbon (PC) and particulate nitrogen (PN) concentrations. Subsamples from the incubated suspended fraction were taken on 12 ml Exetainer® tubes (Labco, Exeter, UK) and the ^15^N atom % enrichment of seawater was measured using a membrane inlet mass spectrometer onboard (MIMS, Hiden HPR40, Warrington, UK). Bulk N_2_ fixation rates were calculated according to White et al.^65^, applying volume corrections for the different MSC fractions as recommended by Riley et al.^41^.

### NifH antibody staining, imaging and cell counts

Particle-associated single-cell N_2_ fixation rates were measured from 500 ml, 50 ml, and 10 ml subsamples taken from each MSC fraction, filtered through 3 µm Isopore polycarbonate filters (Millipore, Tullagreen, Carrightwohill, Ireland). Filters were fixed with 1.6% electron microscopy grade paraformaldehyde (Thermo Fisher, Ward Hill, MA, USA) for 1 h at room temperature, air-dried and stored at −80 °C until further analysis. Samples with significant bulk N_2_ fixation rates were selected for nitrogenase immunolabeling^42^ and nanoSIMS analyses (stations N00, N06, N08, N10 and N13; see below).

Active diazotroph cells were identified and counted by nitrogenase immunolabeling and their contribution to the total bacterial community assessed from comparative DAPI staining. Briefly, cells in filter portions were permeabilised with 0.5% (v/v) dimethyl sulfoxide (DMSO; Sigma-Aldrich, Saint Louis, MO, USA) and washed three times for 5 min in phosphate-buffered saline containing 0.1% Triton X−100 (PBST, Dutscher, Brumath, France), supplemented with 5% (w/v) bovine serum albumin (BSA). Samples were then incubated for 1 h at room temperature in the dark with a polyclonal anti-NifH primary antibody (Agrisera, cat. AS01021A, Vännäs, Sweden) diluted 1:166 (1.2 µl antibody in 198.8 µl PBS). After three rounds of 5 min washes in PBST/BSA, samples were incubated for 45 min at room temperature with a goat anti-Chicken IgY (H + L) secondary antibody conjugated to Alexa Fluor™ 594 (Invitrogen, A-11042), diluted 1:181 (1.1 µl antibody in 198.9 µl PBS). Samples were subsequently washed three times for 5 min in PBST/BSA, and dehydrated in 70% ethanol, and subsequently mounted on slides using 4’,6-diamidino-2-phenylindole (DAPI, ProLong^TM^ glass with DAPI, Thermo Fisher Scientific, Waltham, MA, USA) and stored at −20 °C until visualisation. To test the specificity of the antibody, anti-nitrogenase antibody controls were performed (Supplementary Information).

Samples were visualised by epifluorescence microscopy using a Zeiss Axio Imager.Z2m microscope (Carl Zeiss MicroImaging, Barcelona, Spain) equipped with an AxioCam MRm camera, at 630× magnification. Images were acquired in black and white using the AxioVision software. A Colibri LED light source was used with the HE-62 multi-filter module, including excitation filters BP 370/40 for DAPI and BP 585/35 for Alexa594, a triple beam splitter (TFT 395/495/605), and emission filters TBP 425/46 + 527/54 + LP615. Exposure times were set to 100 ms for DAPI and 120 ms for the Alexa594-labelled antibody. At least ten fields of view were recorded per filter.

Image processing and cell quantification were performed using the automated image analysis software ACMEtool3 (48). Cells were first identified in the DAPI channel and classified as bacterial based on their size (area 18-200 pixels), with an additional signal-to-background ratio (SBR > 2) constraint applied to 0.2 µm filters. NifH-positive (NifH^+^) cells were subsequently identified within the DAPI mask by detecting an AF594 fluorescence signal associated with a DAPI-identified cell. Cells were classified as NifH⁺ only when an AF594 signal overlapped a DAPI-labelled cell and met channel-specific detection thresholds applied during image analysis.

In total, 1,952 fields of view were analysed on 3 µm (particle-associated) filters (95,908 DAPI cells screened, including 3,064 NifH positive cells), and 1753 fields of view on 0.2 µm (free-living) filters (986,147 DAPI cells screened, including 7687 NifH positive cells). No NifH positive cells were detected in the slow sinking fraction; therefore, single-cell mass spectrometry analyses were performed only on the suspended (*n* = 402 cells) and fast sinking (*n* = 327 cells) fractions.

### Single-cell N_2_ fixation rates

Samples displaying the strongest and most spatially coherent NifH signal (i.e., NifH positive) on 3 µm filters (i.e., particle-associated) were selected for nanoSIMS analyses and re-examined by epifluorescence microscopy using an Olympus BX61 microscope equipped with a monochrome Hamamatsu ORCA camera. NifH immunofluorescence was detected using a custom optical cube assembled from Chroma filter components, consisting of an ET599/13x excitation filter, a T612lpxr long-pass dichroic mirror, and an ET632/28 m emission filter (Chroma Technology Corp., Bellows Falls, VT, USA), enabling enhanced isolation of the nitrogenase-associated signal and minimising spectral overlap with Chorophyll *a* fluorescence. DAPI fluorescence was detected using a UB-BP filter set (excitation 360/40 nm, dichroic 400 LP, emission 460/50 nm).

Selected regions from the corresponding filters were then subjected to a freeze-transfer procedure to relocate cells and particles onto a solid substrate compatible with high-resolution analyses. Briefly, filter sections were gently rinsed with Milli-Q water, placed upside down onto patterned silicon wafers (1.2 × 1.2 cm, 1 × 1 mm raster; Pelotec SFG12 Finder Grid substrate, Ted Pella Inc., Redding, CA, USA), rapidly frozen at −80 °C, and carefully removed while frozen to promote transfer of attached material onto the wafer surface^32^. Following transfer, silicon wafers were re-examined using the same epifluorescence microscope and filter sets to relocate NifH-positive particles. The engraved coordinate grid of the silicon substrate was used to map the position of individual target particles, providing precise spatial references for subsequent nanoSIMS analyses. Prior to nanoSIMS measurements, wafers were coated with a gold layer of approximately 30 nm thickness.

The ^15^N atom % enrichment of particle-associated diazotroph cells was measured on a nanoSIMS 50 L (CAMECA, Gennevilliers, France) at the Leibniz Institute for Baltic Sea Research (IOW, Germany). A 1 pA 16 keV Caesium (Cs^+^) primary beam was scanned on a 512 × 512 pixel raster with a raster area of 15 × 15 µm, and a counting time of 250 µs per pixel. Samples were pre-sputtered with 600 pA Cs^+^ current for 2 min in a raster of 30 × 30 µm to remove the gold and surface contaminants and reach the steady state of ion formation. Negative secondary ions ^12^C^-^, ^13^C^-^, ^12^C^14^N^-^, ^12^C^15^N^-^ and ^31^P^-^ were detected with electron multiplier detectors, and secondary electrons were simultaneously imaged. Sixty serial quantitative secondary ion mass planes were generated, drift corrected, and accumulated to the final image. Mass resolving power was >8000 to resolve isobaric interferences. Data was processed using the Look@nanoSIMS software^66^. Lab internal natural abundance samples were used for the adjustment of the nanoSIMS detectors prior to running the enriched samples of the project and it was verified that the natural abundance isotope ratios are reached in Real Time Counting. Isotope ratio images were generated by dividing the ^12^C^15^N^-^ ion count by the ^12^C^14^N^-^ ion count pixel by pixel. Individual diazotroph cells were identified from ^12^C^-^ and ^31^P images. These images were used to define regions of interest (ROIs). For each ROI, the ^15^N/^14^N ratios were calculated based on the ion counts averaged over the ROIs. Additional ROIs were defined in areas without visible ¹⁵N enrichment to estimate internal background ratios, which were centred around natural abundance ( ~ 0.00367; ^15^N atom %). Cells were considered isotopically enriched only when their ¹⁵N/¹⁴N ratios exceeded both natural abundance and the Poisson counting error associated with ion counting statistics. The total number of particles analysed was 729. Single-cell N_2_ fixation rates were calculated according to Furbo Reeder et al.^32^.

### DNA sampling, extraction, *nifH* gene sequencing and bioinformatics

Samples for DNA analyses were sequentially filtered through 3 µm Isopore polycarbonate filters (Millipore, Tullagreen, Carrightwohill, Ireland), and 0.2 µm Supor filters (Pall Corporation, Ann Arbor, MI, USA) to obtain particle-associated and free-living DNA fractions, respectively. Filters were transferred to 2 ml bead beater tubes containing sterile 0.1 mm and 0.5 mm silica beads and stored at -80 °C until DNA extractions ashore.

DNA was extracted from the particle-associated (3 µm) and free-living (0.2 µm) fractions using the DNeasy Plant Mini Kit (Qiagen, Courtaboeuf, France) with additional freeze-thaw bead beating and proteinase-K steps (Scientific Laboratory Supplies, Wilford, Nottingham, UK) before the kit purification step^67^. Triplicate nested PCR reactions were conducted using the degenerate primers nifH3 (5’-ATRTTRTTNGCNGCRTA-3’) and nifH4 (5’-TTYTAYGGNAARGGNGG-3’) in the first PCR, followed by a second amplification with nifH1 (5’-TGYGAYCCNAARGCNGA-3’) and nifH2 (5’-ADNGCCATCATYTCNCC-3’) primers^68,69^ (Eurofins Genomics, Nantes, France). The PCR mix was composed of 5 μl of 5X MyTaq red PCR buffer (Bioline, Memphis, TN, USA), 1.25 μl of 25 mM MgCl_2_, 0.5 μl of 20 μM forward and reverse primers, 0.25 μl Platinum Taq, and 5 μl of DNA extract (1 μl on the second round). The reaction volume was adjusted to 25 μl with PCR-grade water (Invitrogen, Carlsbad, CA, USA). All PCR reagents, Taq polymerase and buffers were purchased from Thermo Fisher (Ward Hill, MA, USA). Triplicate PCR products were pooled and purified using the GeneClean Turbo kit (MP Biomedicals, Santa Ana, CA, USA).

Partial adapters were added by ligation at the AZENTA/Genewiz sequencing facility (Leipzig, Germany) and llumina MiSeq 2 × 300 paired end sequenced. Demultiplexed paired-end sequences were dereplicated, denoised, assembled and chimeras discarded using the DADA2 pipeline^70^. Between 1966 and 227,863 reads were obtained per sample (mean ± SD: 46,745 ± 32,342 reads per sample). In total, 8,451,393 *nifH* gene sequences were obtained resulting in 494 amplicon sequence variants (ASVs; submitted to NCBI under project number PRJNA1412537). ASVs were annotated down to the genus level using a DADA2-formatted *nifH* gene database^71^. ASVs accounting for 1% or more of the reads for at least one of the samples were grouped into 44 genera according to the database (Supplementary Data 2), and those sequences not identified to the genus level were grouped as ‘unknown’ betaproteobacteria, cyanobacteria or deltaproteobacteria. We obtained 8,451,393 sequences and 494 amplicon sequence variants (ASVs), annotated as 37 families and 49 genera (Supplementary Data 3).

### Data analysis, statistics, and plots

All data, statistical analyses and plots were done in RStudio v2024.04.2^72^. All plots were done using the *ggplot2*^73^ package, except CTD plots that were done using the *oce*^74^ R package. N_2_ fixation values were checked for normality using a Shapiro-Wilk test and significant differences between samples tested with Wilcoxon test using the *dplyr* package^75^. Amplicon data heatmaps plotted with the *ampvis2* R package^76^. Redundancy analysis biplots were calculated and plotted with the *phloyseq* R package^77^. Missing values in the environmental metadata were imputed prior to redundancy analysis to avoid excessive sample loss. Remaining missing entries in the mixed-type metadata set were imputed using the random forest-based algorithm implemented in the *missForest* package in R^78^, with a fixed random seed (123) to ensure reproducibility. The imputed metadata matrix (Supplementary Data 5) was subsequently used as the sample-data component when constructing the *phyloseq* object for ordination analyses.

## Supplementary information

Supplementary Information

Peer Review file

Description of Additional Supplementary Files

Supplementary Data 1

Supplementary Data 2

Supplementary Data 3

Supplementary Data 4

Supplementary Data 5

## Data Availability

Source data is provided with this paper. Water mass classification is available at 10.5281/zenodo.18619607. Stainless steel and titanium cast data are available at 10.5285/48e39c93-2405-99f3-e063-7086abc003fa and 10.5285/48c1d94c-1647-8717-e063-7086abc0f520, respectively. *nifH* gene sequences are available at NCBI under project number PRJNA1412537.
