# Supplementary material for: Particle-associated diazotrophs drive nitrogen fixation in Arctic subsurface waters of the Barents Sea
Source: Nat Commun. 2026 Jul 8;17:8417. doi: 10.1038/s41467-026-75354-5 (PMC13478305; doi:10.1038/s41467-026-75354-5)
Supplement: Supplementary file 1 [file 41467_2026_75354_MOESM1_ESM.pdf]

Supplementary Information

Particle-associated diazotrophs drive nitrogen fixation in Arctic subsurface waters

Mar Benavides, Arthur Coët, Marta Sebastián, Angela Vogts, Christian Furbo Reeder, Elena Cerdán-García, Louisa Norman, Ruth Hawley, Oliver Flanagan, Maeve C. Lohan, Joanne E. Hopkins, Claire Mahaffey

Mar Benavides
Email: mar.benavides@noc.ac.uk

Includes:
Supplementary Methods
Supplementary Results
Figures S1-S8
Tables S1-S5

Supplementary Methods

Biogeochemical and hydrographic data

The DY167 N-ARC GEOTRACES GApr19 cruise took place from 9 July to 13 August 2023 onboard the RRS *Discovery.* Water column profiles of pressure, temperature, salinity were obtained with a Seabird SBE 911plus CTD along with fluorescence, beam transmission, turbidity, oxygen and photosynthetic active radiation (PAR) sensors mounted on a 24-Niskin bottle stainless steel rosette frame.

Nutrient concentrations (nitrate plus nitrite = NN and phosphate = PO4 were determined onboard from triplicate fresh filtered samples using a Bran and Luebbe QuAAtro 5-channel nutrient autoanalyzer [1,2]. The limit of detection of NN and PO4 was 0.1 µM. CTD fluorescence, oxygen and salinity profiles were calibrated against experimental values measured from seawater samples collected from stainless stell CTD casts as described in the DY167 cruise report [3]. Dissolved and particulate iron (dFe and pFe, respectively) concentrations were obtained from seawater samples collected with Go-Flo bottles mounted on a titanium rosette frame following GEOTRACES protocols [3,4]. Stainless steel and titanium cast data are available in references [5] and [6], respectively.

An optimum multi-parameter (OMP) analysis was performed to identify the fractions of five key regional water masses present at sampling stations: Atlantic Water (AW), warm Atlantic Water (wAW), Arctic Water (AW), warm Arctic Water (wArW) and Glacial Water (GW) [7]. Both physical and biogeochemical tracers were used to constrain the results: conservative temperature, absolute salinity, nitrate+nitrite, silicate, and the meteoric and sea ice melt fractions derived from oxygen isotope samples [7]. For each station, the MSC sampling depths were matched to the nearest OMP result to provide an indication of the dominant water pass present.

Nitrogenase antibody immunolabeling specificity

Nitrogenase (NifH) immunolabeling specificity was verified using complementary negative controls (Fig. S7). Full immunolabeling performed on a non-diazotrophic strain (*Pseudomonas guineae*; Fig. S7A) did not yield any detectable signal in the NifH (Alexa Fluor 594) channel. Similarly, omission of the primary antibody on the diazotrophic strain *Vibrio diazotrophicus* resulted in no detectable NifH signal (Fig. S7B). Finally, primary-antibody omission controls performed on marine snow particle samples collected on 3 µm filters also showed no detectable fluorescence in the NifH channel (Fig. S7C). Together, these controls confirm the absence of non-specific antibody binding and demonstrate that the secondary antibody alone does not generate detectable background signal.

^{15}N signal dilution considerations

Staining procedures such as CARD-FISH or immunolabeling can dilute the isotopic enrichment of samples, underestimating isotope uptake rates [8]. Considering the nitrogen content of the different chemicals used for antibody staining and considering the ^{15}N introduced into our sample (quantified by MIMS), we estimated the potential dilution of the signal. Sample were incubated in a blocking solution containing 5% (w/v) BSA (50 mg ml^{-1} of protein), and exposed to primary and secondary antibodies (1.2 µl and 1.1 µl in 200 µl, respectively), which represent a negligible contribution compared to BSA. We assume that all these compounds contain nitrogen at natural isotopic balance (0.0366% ^{15}N). Only a small fraction of this bulk protein can interact with cells. Based on reported adsorption rates (0.05-1 µg cm^{-2}, up to 2.5 µg cm^{-2}; [9]) and a bacterial surface around 3×10^{-8} cm^{-2} (Supplementary Data 3), this corresponds to 1.5 fg of adsorbed protein, or 0.24 fg N per cell (assuming 16% nitrogen content of proteins). Given that a representative cell contains 100 fmol N (Supplementary Data 3), a highly conservative worst-case scenario, assuming complete incorporation and mixing of this external nitrogen, yield a maximum dilution of 15%. This estimate in intentionally conservative, as it neglects washing steps and assumes full

intracellular incorporation of surface-bound proteins, which is unrealistic since proteins mainly remain adsorbed at the cell surface and are largely removed during washing. In practice, adsorption is largely restricted to the cell surface, and the actual dilution is expected to be substantially lower. This upped bound estimate is consistent with the dilution values reported by Musat et al. [8] for CARD-FISH applications.

Comparison of particle-associated diazotroph communities with lower latitude studies

To allow a direct comparison of particle-associated diazotroph communities across cruises and with lower-latitude studies, raw *nifH* amplicon sequences from the three campaigns ([10,11] and this study) were reprocessed jointly using a unified bioinformatic pipeline based on DADA2 [12]. All raw FASTQ files were pooled and treated together to infer a single, shared set of amplicon sequence variants (ASVs), thereby avoiding inconsistencies arising from independent denoising.
Both paired-end and forward-only reads were included. For paired-end reads, forward and reverse sequences were quality-filtered using filterAndTrim with truncation lengths of 230 bp (forward) and 150 bp (reverse), removal of reads containing ambiguous bases (maxN = 0), a maximum expected error threshold of 2 for each read (maxEE = c(2,2)), truncation at a quality score of 2 (truncQ = 2), primer trimming of 17 bp on both reads (trimLeft = c(17,17)), and removal of PhiX reads. Forward-only reads were processed separately using the same quality-filtering criteria, except for a truncation length of 180 bp and primer trimming of 17 bp on the forward read only. Error models were learned jointly across all filtered reads. Paired-end reads were denoised independently for forward and reverse directions and subsequently merged, while forward-only reads were denoised up to ASV inference. ASV tables derived from paired-end and forward-only datasets were then merged into a single sequence table. Chimeric sequences were removed using the consensus method implemented in DADA2.

ASVs were anotated using a curated *nifH* database [13] Taxonomy was assigned using the DADA2 assignTaxonomy function with a minimum bootstrap confidence threshold of 80. This unified processing ensured that differences observed between cruises reflect biological variation rather than methodological biases. Family-level *nifH* ASVs were Hellinger-transformed and NMDS-ordained on Bray-Curtis dissimilarities using the *microViz* R package [14]. Overlap of *nifH* ASVs among ocean basins was visualised using a three-way Venn diagram. ASV presence/absence was determined separately for each ocean basin (Pacific: NCDC cruise [10]; Atlantic: e-IMPACT cruise [15]; Arctic: NARC cruise: this study). ASVs were considered present in an ocean basin if they occurred in at least one sample from that cruise. Overlap sizes represent raw counts and percentages (relative to total ASVs across all three basins) and were generated using the *VennDiagram* R package [16].

Supplementary Results

Environmental gradients sampled

Hydrographic (CTD casts) and biogeochemical (macro- and micronutrients) data [6] were collected across the full range of environmental conditions and water mass gradients in the Barents Sea (Fig. 1), described in Hawley et al. [17]. This included warm, saline Atlantic Water (AW) inflow; cold, fresh Arctic Water (ArW) north of the Polar Front at 76°N; the sea-ice edge; and regions influenced by glacial melt (Supplementary Data 1). With the exception of the shallow Spitzbergen Bank (station N12), the whole sampling area was seasonally stratified, resulting in a well-mixed, nutrient depleted, surface layer of either warm Atlantic Water (wAW) or warm Arctic water (wArW). On the shelf, the strength of stratification doubled between AW dominated stations south of the Polar Front (stations N03 to N05) and those at the ice-edge (in ArW) where sea-ice melt had introduced a thin, fresh surface water cap (stations N08 and N09). Coincidently the depth of the surface mixed layer decreased with latitude, from 23-27 m at stations N03-N05 to 6-11 m at stations N08-N09. This was mirrored by a deepening of the subsurface chlorophyll maximum from an average of 28 m at stations N03-N05 (at the base of the surface mixed layer), to 61 m at stations N08-N09 (significantly below the surface mixed layer).

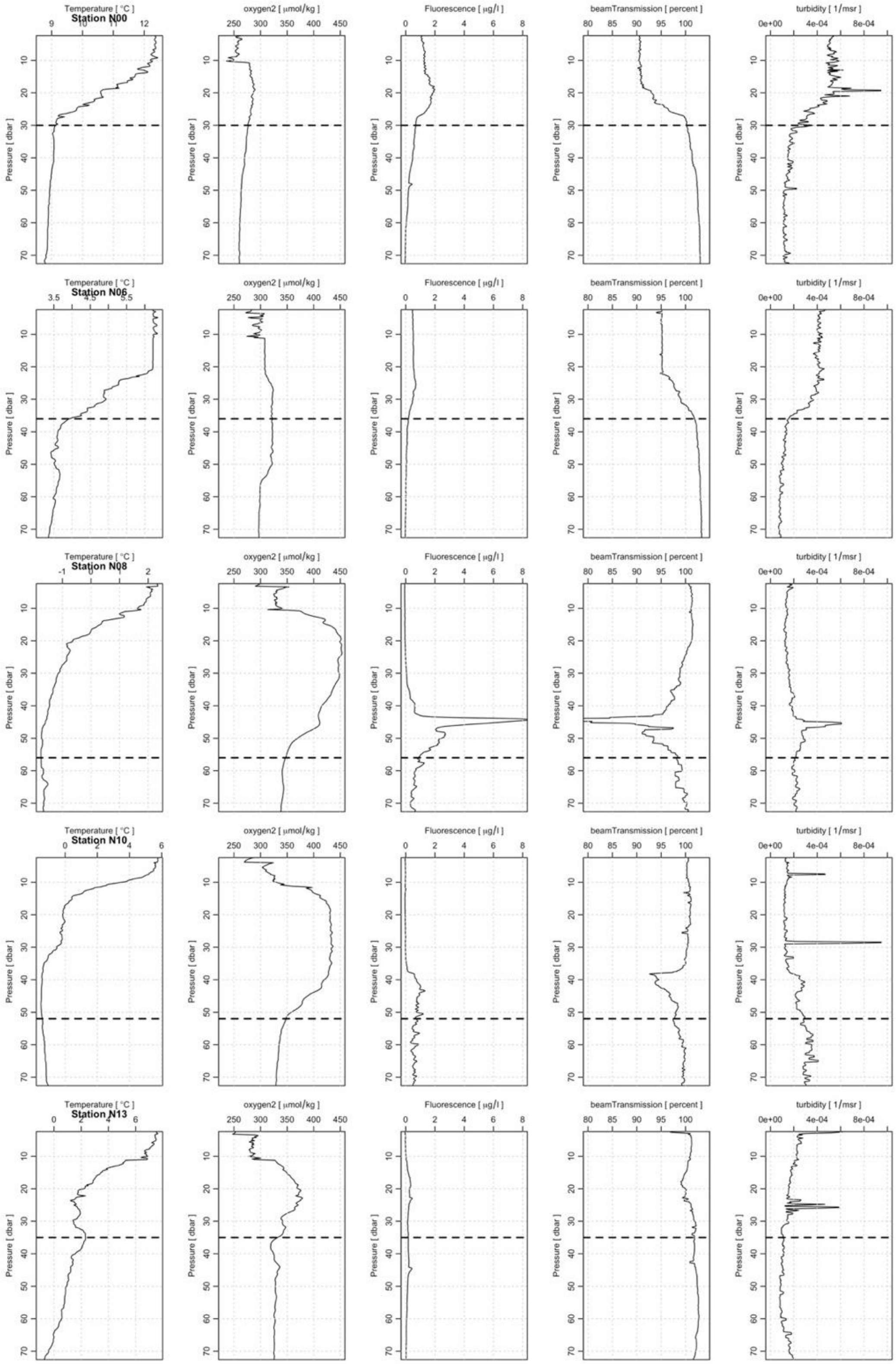

Fig. S1. Hydrographic profiles. Temperature, oxygen, fluorescence, beam transmission and turbidity profiles at stations where single-cell N_2 fixation measurements were performed (i.e., stations N00, N06, N08, N10 and N13). Dashed lines indicate the depth at which the marine snow catcher (MSC) was deployed.

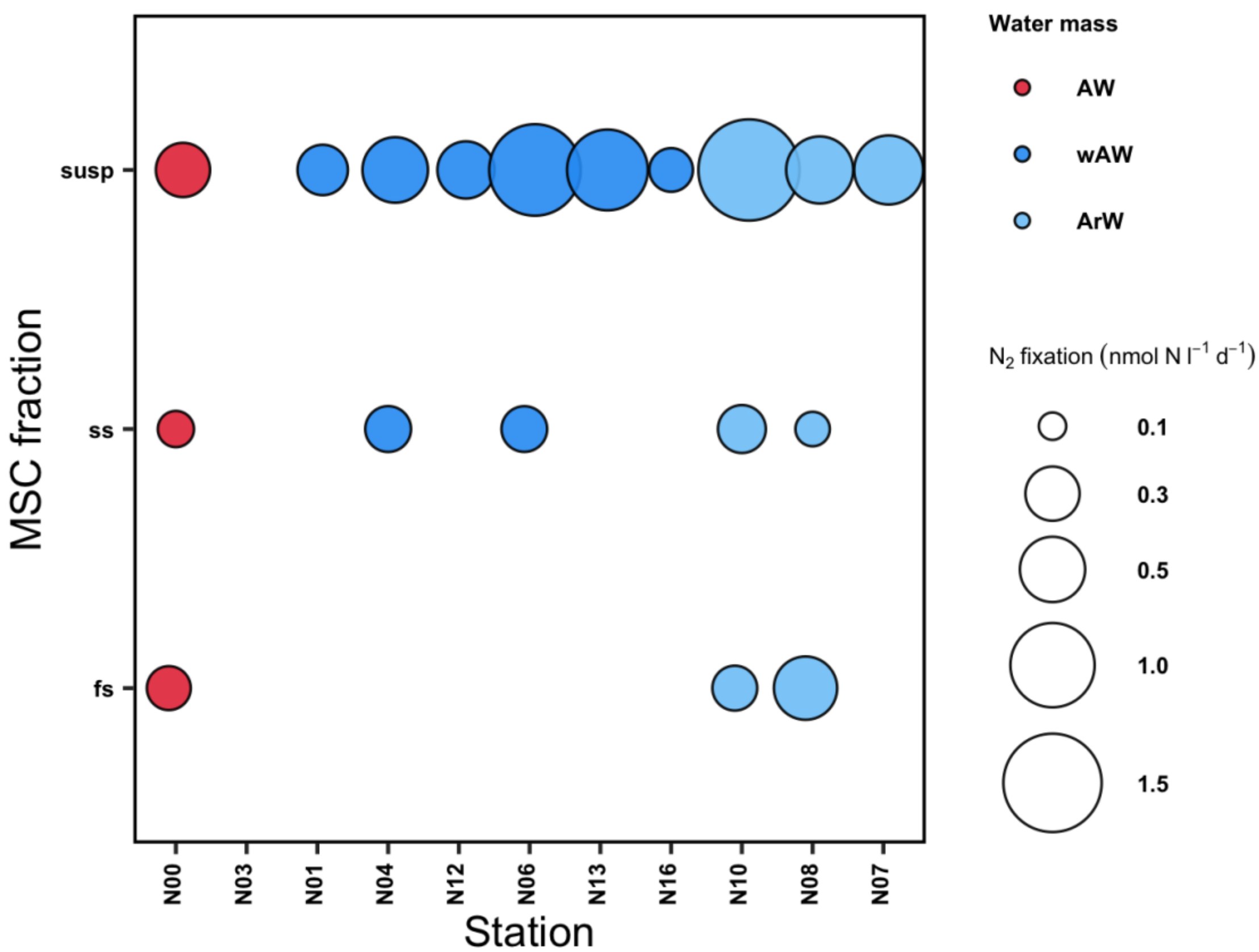

Fig. S2. Bulk N_2 fixation rates in MSC fractions. Bulk N_2 fixation rates in the suspended (susp), slow sinking (ss) and fast sinking (fs) marine snow catcher (MSC) particle fractions, coloured by water mass (AW = Atlantic Water n = 5, wAW = warm Atlantic Water n = 14, ArW = Arctic Water n = 8). The size of the circles depicts the magnitude of the rate. Average and standard deviation values are shown in Supplementary Data 5.

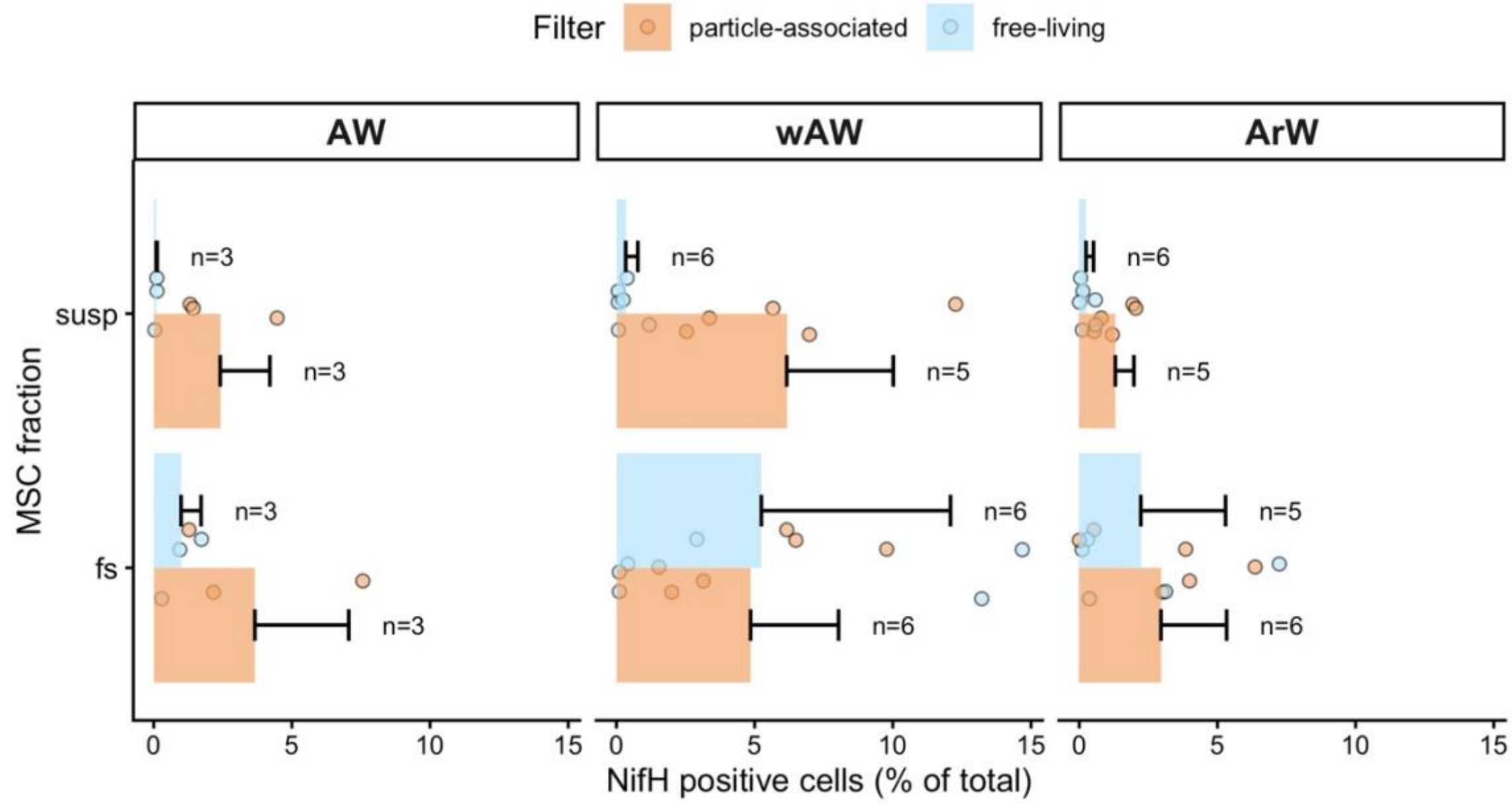

Fig. S3. Counts of particle-associated and free-living diazotrophs. Average percentage contribution of free-living (0.2 µm filters) and particle-associated (3 µm filters) diazotrophs (DAPI and NifH positive cells) to the total microbial community (DAPI positive cells). Percentage contributions are faceted by water mass (AW = Atlantic Water, wAW = warm Atlantic Water, ArW = Arctic Water) and marine snow catcher particle fraction (susp = suspended, ss = slow sinking, fs = fast sinking). NifH antibody positive cells in the ss fraction were only detected in wAW waters and were not further considered for nanoSIMS analyses (see Fig. 2).

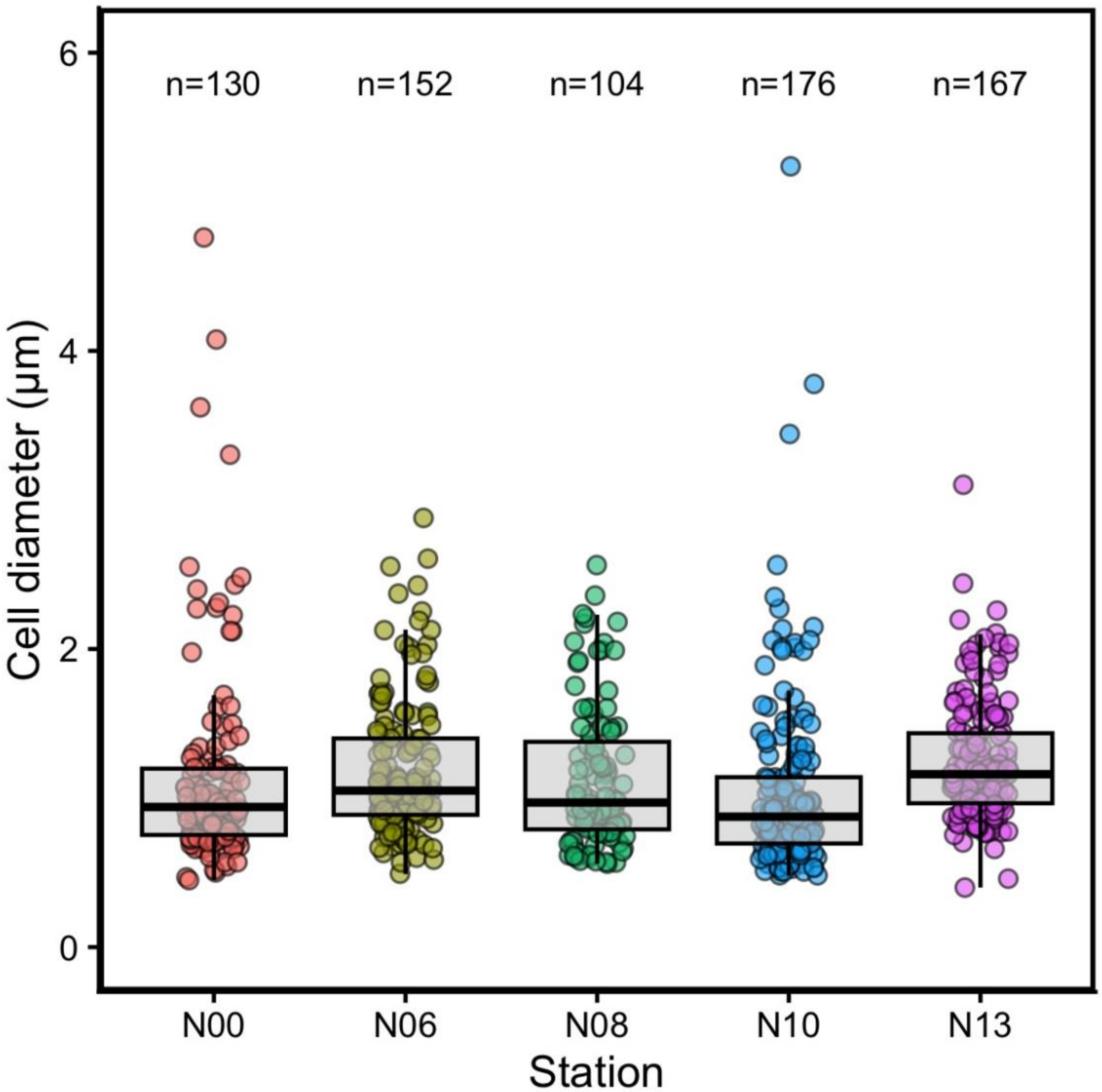

Fig. S4. Diameter of particle-associated cells. Diameter of particle-associated NifH and DAPI positive cells used for nanoSIMS analyses by station.

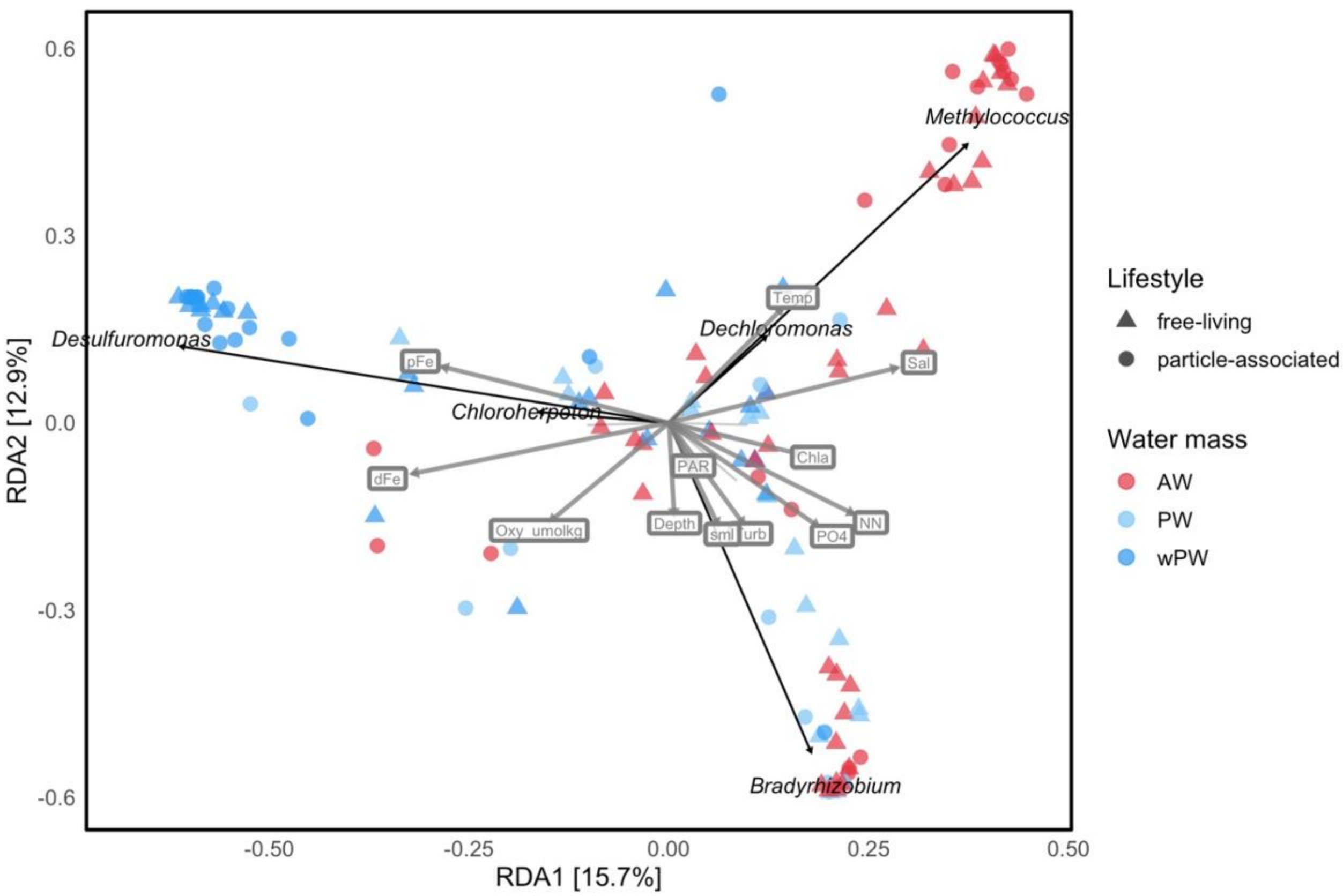

Fig. S5. Multivariate redundancy analyses of diazotroph communities and metadata. Multivariate redundancy analysis (RDA) biplot depicting the variance of *nifH* gene sequences explained by the environmental parameters and diazotroph genera grouped by water mass in colours (AW = Atlantic Water, wAW = warm Atlantic Water, ArW = Arctic Water). Explanatory variable vectors include depth = Depth, temperature = Temp, salinity = Sal, dissolved oxygen = Oxy_umolkg, chlorophyll *a* = Chla, photosynthetic active radiation = PAR, turbidity = Turb, nitrate + nitrite = NN, phosphate = PO4, dissolved iron = dFe, particulate iron = pFe, and surface mixed layer depth = SML.

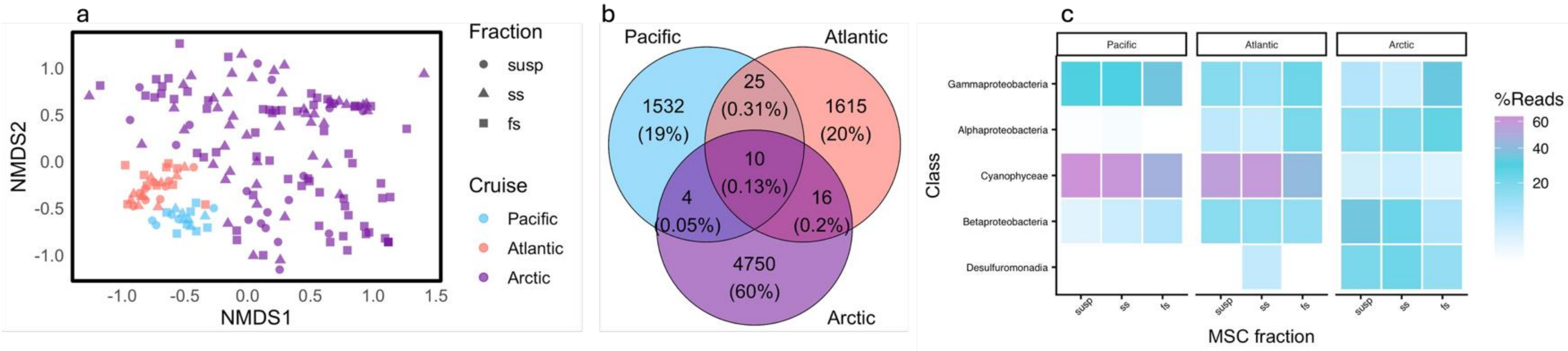

Fig. S6. Comparison of marine snow catcher derived diazotroph communities across the Pacific, Atlantic and Arctic ocean regions. *nifH* amplicon sequences variants (ASVs) from this study (Arctic, N-ARC cruise) and other published studies from the North Pacific (NCDC cruise) [10] and North Atlantic (eIMPACT cruise) [11] obtained using marine snow catcher (MSC) deployments.(a) Non-metric multidimensional scaling (NMDS) analysis distinguishing between MSC fractions with susp = suspended, ss = slow sinking and fs = fast sinking (shape) and cruise (colour); (b) Venn diagram showing percentage overlap of ASVs among the three studies; (c) Heatmap showing the $\log_{10}$ *nifH* gene relative abundance of all the ASVs annotated at the Class level, faceted by cruise MSC fractions.

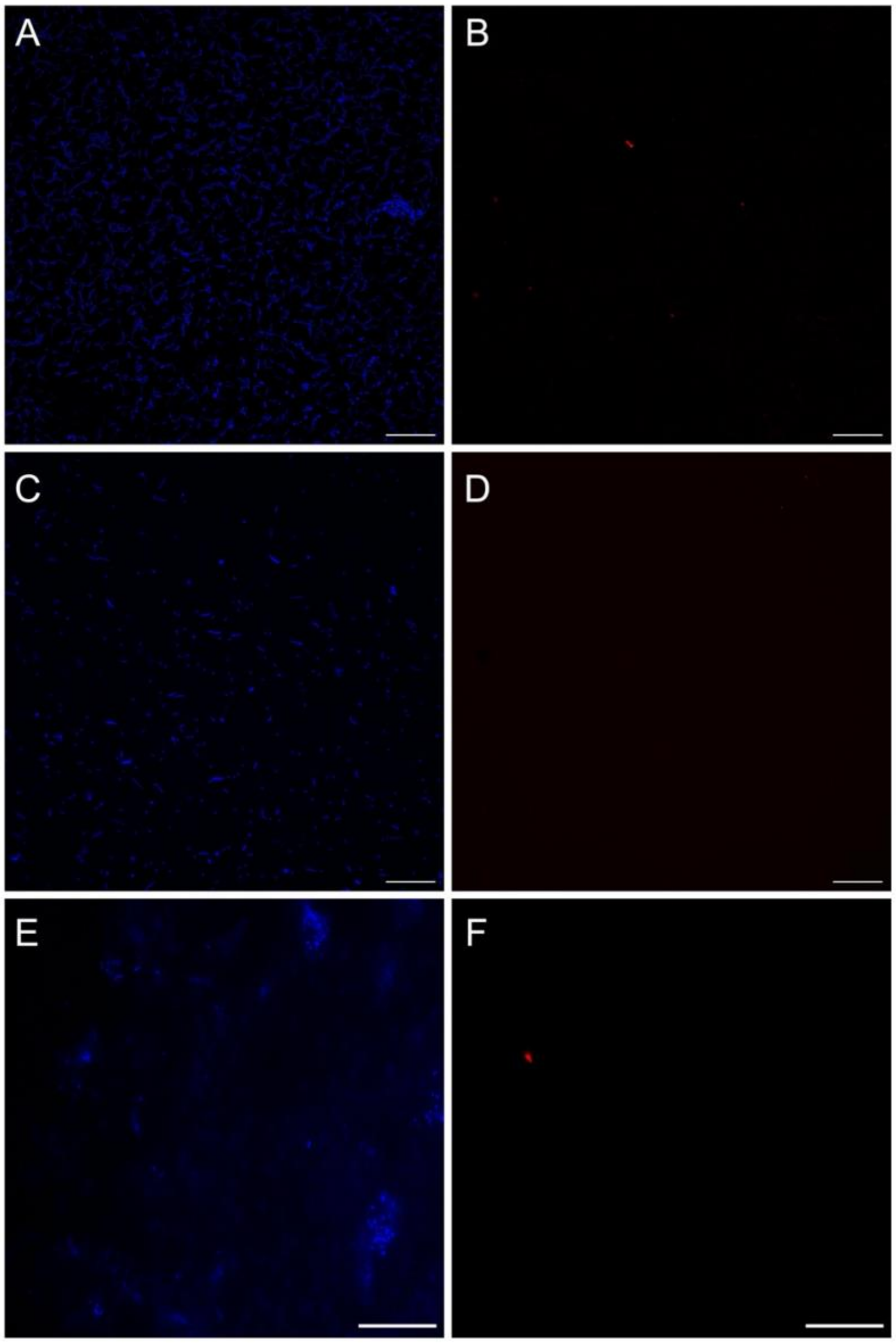

Fig. S7. Immunolabeling specificity tests. (A) DAPI-stained image of *Pseudomonas guineae*, showing all bacterial cells. (B) Fluorescence image of *P. guineae* following the complete nitrogenase immunolabeling protocol and acquired under excitation of the fluorochrome coupled to the anti-nitrogenase antibody. As *P. guineae* is a non-diazotrophic bacterium, no specific cellular fluorescence is detected, only sparse background artifacts are visible. (C) DAPI-stained image of *Vibrio diazotrophicus*, showing all bacterial cells. (D) Fluorescence image of *V. diazotrophicus* acquired under excitation of the secondary antibody-coupled fluorochrome, with omission of the primary anti-nitrogenase antibody. The absence of cellular fluorescence indicates that the secondary antibody does not bind non-specifically. (E) DAPI-stained image of the particle-attached fraction collected during the N-ARC cruise and retained on a 3 µm filter, showing aggregates of small bacterial cells associated with marine particles. (F) Fluorescence image of the same particle-attached fraction under NifH/Alexa excitation, with omission of the primary anti-nitrogenase antibody. No specific fluorescence is detected, confirming the absence of non-specific binding; only occasional imaging artifacts are observed.

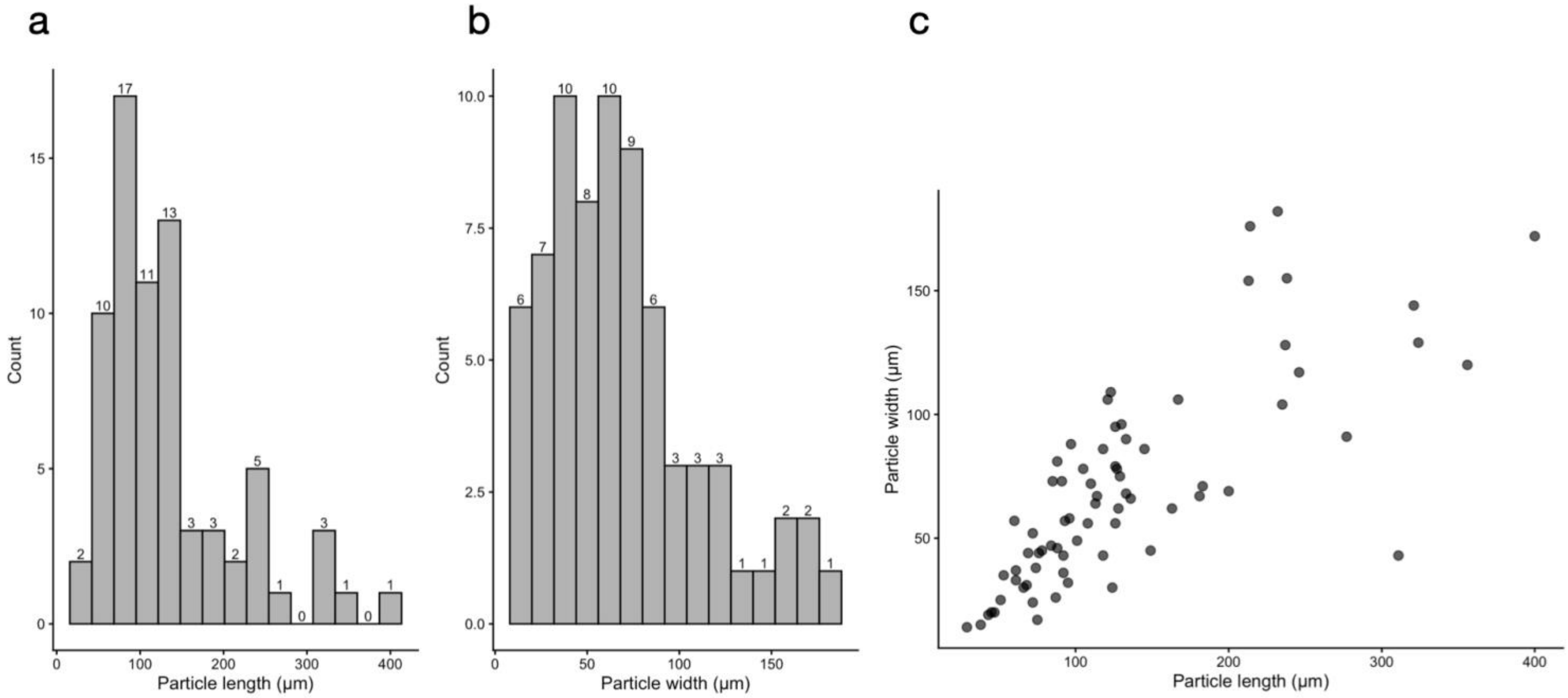

Fig. S8: Particle dimensions. Distribution of particle lengths (a) and widths (b) measured from microscopy images using the major and minor particle axes, respectively, of particles collected on 3 µm filters (n = 72 particles measured across 10 filter samples). Numbers above histogram bars indicate the number of particles within each size bin. Panel (c) shows the relationship between particle length and width. Particle length ranged from 29 to 400 µm (132.9 ± 80.2 µm, mean ± SD), while particle width ranged from 14 to 182 µm (68.0 ± 40.5 µm, mean ± SD).

Supplementary Data 1. Sampling station metadata. List and classification of sampled stations and type of sampling conducted at each. Seawater mass classification was based on ref [16].

Supplementary Data 2. Bulk N_2 fixation rates. Bulk N_2 fixation rates by marine snow catcher (MSC) fractions, station number, and water mass (AW = Atlantic Water, wAW = warm Atlantic Water, ArW = Arctic Water).

Supplementary Data 3. Particle-associated N_2 fixation rates. ^{15}N atom % enrichment, cell diameter, biovolume, particulate carbon (PC), particulate nitrogen (PN) and N_2 fixation rates of single-cells measured from 3 µm filters on suspended (susp) and fast sinking (fs) marine snow catcher (MSC) fractions.

Supplementary Data 4. Amplicon sequence variants. Taxonomic annotation of amplicon sequence variants (ASVs).

Supplementary Data 5. Metadata used in multivariate redundancy analysis. Environmental metadata used in redundancy analysis (RDA, Fig. S5).

SI References

1. Brewer, P. G. & Riley, J. P. The automatic determination of nitrate in sea water. *Deep Sea Res. Oceanogr. Abstr.* **12**, 765–772 (1965).
2. *Methods of Sea-Water Analysis*. (Wiley-VCH Verlag, Weinheim, Germany, 1999).
3. Hopkins, J. E. & Mahaffey, C. *Nitrogen Fixation in the Arctic N-Arc DY167 Cruise Report GEOTRACES GApr19*. https://www.bodc.ac.uk/resources/inventories/cruise_inventory/reports/dy167.pdf (2023).
4. Cutter, Gregory, Casciotti, Karen, Croot, Peter, Geibert, Walter, Heimbürger, Lars-Eric, Lohan, Maeve, Planquette, Hélène. *Sampling and Sample-Handling Protocols for GEOTRACES Cruises*. https://repository.oceanbestpractices.org/handle/11329/409 (2017).
5. Mahaffey, C. *et al.* Stainless Steel CTD water column samples (macronutrients, chlorophyll-a, particulate carbon, C-fixation, d18O) and upcast sensor measurements (temperature, salinity, oxygen, optical backscatter, beam transmittance and attenuation, PAR, fluorescence) from cruise DY167 (GApr19), July-August 2023. (2026).
6. Lohan, M. *et al.* Titanium CTD water column samples (macronutrients, chlorophyll-a, trace elements) and upcast sensor measurements (temperature, salinity, oxygen, optical backscatter, beam transmittance and attenuation, PAR, fluorescence) from the Norwegian and Barents Sea on cruise DY167 (GApr19), July-August 2023. https://doi.org/10.5285/48c1d94c-1647-8717-e063-7086abc0f520 (2026).
7. Ilyas, M., Hopkins, J., Mahaffey, C. & Norman, L. Classification of Barents Sea water masses from cruise DY167 (RRS Discovery) in July-August 2023 using Optimum Multiparameter (OMP) analysis. Zenodo https://doi.org/10.5281/ZENODO.18619607 (2026).
8. Musat, N. *et al.* The effect of FISH and CARD-FISH on the isotopic composition of (13)C- and (15)N-labeled Pseudomonas putida cells measured by nanoSIMS. *Syst. Appl. Microbiol.* **37**, 267–276 (2014).
9. Guha Thakurta, S. & Subramanian, A. Evaluation of in situ albumin binding surfaces: a study of protein adsorption and platelet adhesion. *J. Mater. Sci. Mater. Med.* **22**, 137–149 (2011).

10. Furbo Reeder, C. *et al.* Unveiling the contribution of particle-associated non-cyanobacterial diazotrophs to N_2 fixation in the upper mesopelagic North Pacific Gyre. *Commun. Biol.* (2025).
11. Cerdán-García, E., Álvarez-Salgado, X. A., Arístegui, J., Martínez-Marrero, A. & Benavides, M. Eddy-driven diazotroph distribution in the subtropical North Atlantic: horizontal variability prevails over particle sinking speed. *Commun. Biol.* **7**, 929 (2024).
12. Callahan, B. J. *et al.* DADA2: High-resolution sample inference from Illumina amplicon data. *Nat. Methods* **13**, 581–583 (2016).
13. Morando, M. *et al.* Global biogeography of N_2-fixing microbes: nifH amplicon database and analytics workflow. *Earth System Science Data Discussions* 1–39 (2024) doi:10.5194/essd-2024-163.
14. Barnett, D., Arts, I. & Penders, J. microViz: an R package for microbiome data visualization and statistics. *J. Open Source Softw.* **6**, 3201 (2021).
15. Chen, H. & Boutros, P. C. VennDiagram: a package for the generation of highly-customizable Venn and Euler diagrams in R. *BMC Bioinformatics* **12**, 35 (2011).
16. Hawley, R. *et al.* Iron and manganese cycling in the atlantifying Barents Sea: Concentrated inputs and emerging limitations. *Global Biogeochem. Cycles* **40**, e2025GB009031 (2026).
17. Sundfjord, A. *et al.* Suggested water mass definitions for the central and northern Barents Sea, and the adjacent Nansen Basin. *The Nansen Legacy Report Series* (2020) doi:10.7557/nlrs.5707.
