# Supplementary material for: Particle-associated diazotrophs drive nitrogen fixation in Arctic subsurface waters of the Barents Sea
Source: Nat Commun. 2026 Jul 8;17:8417. doi: 10.1038/s41467-026-75354-5 (PMC13478305; doi:10.1038/s41467-026-75354-5)
Supplement: Supplementary file 2 [file 41467_2026_75354_MOESM2_ESM.pdf]

nature portfolio

Peer Review File

Particle-associated diazotrophs drive nitrogen fixation in Arctic subsurface waters of the Barents Sea

Corresponding Author: Dr Mar Benavides

This file contains all reviewer reports in order by version, followed by all author rebuttals in order by version.

Version 0:

Reviewer comments:

Reviewer #1

(Remarks to the Author)
Benavides et al shows that particle-associated microbes performed nitrogen fixation in the Atlantic Arctic, and those diazotrophs are non-cyanobacterial. I agree that this result is in itself a novel finding. However, I could not understand how impactful this discovery is.

Recent studies have already shown that particle-associated microbes perform nitrogen fixation in the ocean (Harding et al., 2022). Therefore, I am not particularly surprised that similar lifestyle diazotrophs also exist in the Arctic. For this reason, simply demonstrating the occurrence of nitrogen fixation by particle-associated diazotrophs may not be sufficient.

Another point of concern is that the observation is limited to the Atlantic Arctic and is based on only a single cruise. How is the representativeness of this observation ensured? Based only on the title and abstract, readers may be left with the impression that this phenomenon occurs throughout the Arctic Ocean, but is that really justified? Environmental conditions differ substantially between the Atlantic and Pacific sectors of the Arctic because of differences in water mass inflow, and ecosystem responses also differ between these regions (Ardyna and Arrigo, 2020; Waite et al., 2026). I am not convinced that the authors fully appreciate this point. The Barents Sea, for example, is a region where mixing has intensified owing to the inflow of Atlantic Water, and primary production has correspondingly increased (Ingvaldsen et al., 2021: Noh et al., 2024). Because this production is driven mainly by nitrate supply, I question whether nitrogen fixation would in fact be an important process in such a region.

References
Ardyna, M., Arrigo, K.R. (2020) Phytoplankton dynamics in a changing Arctic Ocean, Nature Climate Change 10, 892-903.
Waite, A.M. et al. (2026) A quantitative comparison of the physical supply and biological uptake of new nitrogen in the Arctic Ocean, Nature reviews earth & environment, https://doi.org/10.1038/s43017-026-00769-z
Ingvaldsen, R.B. et al. (2021) Physical manifestations and ecological implications of Arctic Atlantification, Nature reviews earth & environment 2, 874-889.
Noh, K. et al. (2024) Role of Atlantification in enhanced primary productivity in the Barents Sea, Earth's Future 21, e2023EF003709.

Reviewer #2

(Remarks to the Author)
This brief paper reports bulk nitrogen fixation rates from size fractionated samples collected from 14 stations in the Arctic subsurface (10 m below the chlorophyll max). A subset with the highest bulk rates were then analyzed for single cell N2 fixation. Solid paper, needs a few edits (see comments below)

Comments:
1) I found the use of the acronyms (susp, ss, fs, AW, wAW, ArW) in such a short paper to be tedious (it requires the reader to use their memory trying to remember what the acronyms are as they are trying to read and understand the paper). My suggestion is to avoid the acronyms as much as possible.
2) It does not appear that the authors used formalin-killed controls to determine the background isotope signal. It would be

nice if the authors could state why they feel they do not require this, and if so, how they set up the nanoSIMS detectors to make sure the isotope ratios are correct. For example, do they run a natural abundance control sample first prior to running the isotope labeled samples?
3) Please discuss the size of the particles that were analyzed by nanoSIMS and convince the reader that topography was not an issue that could have resulted in incorrect isotope analyses (nanoSIMS works best on flat samples).
4) There is no discussion about the immunolabeling causing isotope dilution, therefore the N2 fixation rates reported are likely underestimates. Please address this somewhere.
5) Line 139: could you also have used chlorophyll or phycocyanin autofluorescence to determine if those cells were cyanobacteria?
6) Lines 147, 154 mention ASVs, but the main methods have nothing about 16S rRNA gene amplicon sequencing, perhaps a brief statement in the methods that the authors did 16S sequencing and refer the reader to the supplementary.
7) Reading the main paper, I thought that nitrogen fixation was not measured in the free-living fraction (and it seemed to be ignored). But then I saw figure S3. It would be helpful to call this out specifically in the main paper. Also, how are there such high levels of free-living N2 fixers in the wAW fs samples? Aren't those supposed to be mostly particles? This needs a bit of an explanation.
8) Fig 2B: the scale car of the isotopes is too small to see, and it would be helpful for the reader to have the fluorescence and isotope images be at the same scale.
9) Fig 3: why is it necessary to report the free-living microbial community identity if that fraction was not analyzed for N2 fixation? A short statement is enough.
10) Fig. S2: most of the N2 fixation is in the suspended fraction. Is most of that activity in the particles or in the free-living fraction?

Reviewer #3

(Remarks to the Author)
Benavides and colleagues measured nitrogen fixation in the Arctic, focusing specifically on nitrogen fixation on particles. The study uses immunolabelling of the NifH enzyme combined with nanoSIMS to measure 15N isotope incorporation at the single-cell level. The authors compare particles from different water masses as well as particles with different sinking rates, including suspended, fast-sinking, and slow-sinking particles. The study finds particle-associated nitrogen fixation may be a significant source of nitrogen in the Arctic and contributes the observed nitrogen fixation to non-cyanobacteial diazotrophs. Overall, the manuscript is well organized, and the study provides novel data that fills a necessary gap in our understanding of nitrogen fixation and diazotrophs in the Arctic. However, I believe the manuscript could be improved by providing additional context, such as bulk whole water N2 fixation rates and the subsequent estimations of how much particulate N2 fixation contributes to the whole water values. Also, there are single-cell nitrogen fixation rates that are extremely high, without explanation or context for these values, it is plausible that these values are errors associated with the method. Further calculation of assimilation percent, which is used to estimate newly synthesized biomass based on isotope incorporation, would shed light on the biomass turnover behind the high nitrogen fixation rates (Popa et al., 2007).
Below are some additional comments and questions regarding the text.
Line 46: nitrogen, not nutrient
Line 117: is this percentage of the whole microbial community or the community present on particles?
Line 118: wAW waters, waters seems redundant based on the abbreviation
Line 124: how is 1192 fmol N cell-1 day-1 possible? That may be over 100% enrichment and considering the immunolabeling would dilute the isotope, it would in theory be even higher. Hypotheses for these values would be appreciated here or reference to discussing them later in the text.
Line 127: In all water masses, the fs particles were higher than the susp particles? In line 100 the author says susp fraction had the highest N fix rates. Please address for clarity here.
Line 148-152: Can the presence of chlorophyll by epifluorescence be used to verify if cyanobacteria were present? If not, why?
Line 154: top ten nifH AVSs. Also, was sequencing done on whole water samples or particle fractions and free-living fractions? So top 10 ASVs in what? A quick overview of the sequencing strategy here would help set up this paragraph from understanding.
Line 158: again what fraction was Desulfuro abundant?
Line 168: I don't see the connection of the ice-laden sediment transport to the above reference to terrestrial origins, please expand your explanation.
Line 177: The argument in this line is unclear
Line 184: tparticle, remove t
Line 186: Are the sequencing data from the Pacific and Atlantic part of this study or others?
Line 221: Did the 48-hour incubation cause more of the susp fraction to settle? Also, in all the fractions, it seems like settling over 48-hour incubations would create a larger space of particulate matter and potentially more anoxic zones that would not necessarily reflect the environment of sinking particle and would therefore affect the Nfix rates, potentially increasing the rates due to the incubation conditions.
Line 300: If Chlorophyll fluorescence was present, can it be used to identify cyanobacterial versus NCD cells
Line 569: The presence of NifH and DAPI does not necessarily indicate active N2 fixation; the incorporation of 15N does, however.

References
Popa, R. et al. Carbon and nitrogen fixation and metabolite exchange in and between individual cells of Anabaena oscillarioides. ISME J 1,354–360 (2007).

Version 1:

Reviewer comments:

Reviewer #1

(Remarks to the Author)
My impression has not changed after reading their rebuttal letter and revised manuscript. It may indeed be true that this is the first study to observe nitrogen fixation in particles in the Arctic Ocean. However, the importance of Harding et al. (2022) lies in the fact that they were the first to directly measure nitrogen fixation activity in particles. Even before that study, it was already known that organisms with the potential for nitrogen fixation could occur in particles. The value of Harding et al. (2022) was that they demonstrated, for the first time, that nitrogen fixation activity actually occurs within particles.

If diazotrophs were predominantly particle-associated across the pan-Arctic Ocean, this would represent an interesting ecological pattern distinct from those observed in other oceanic regions. However, the available evidence does not support such a broad interpretation. In the Pacific sector of the Arctic Ocean, UCYN-A has been shown to actively contribute to nitrogen fixation (Harding et al., 2018), as the authors themselves seem to acknowledge in their rebuttal letter. Thus, the authors' observations are better interpreted as a local phenomenon occurring in part of the Arctic Ocean, rather than as a general feature of Arctic nitrogen fixation.

I do not see serious flaws in the authors' analytical methods. My main concern is that the authors appear to draw conclusions that go beyond what their results can support. This tendency is also evident in the framing of the Introduction.

A study conducted in the Barents Sea should not be framed as representing the pan Arctic Ocean. Although the Abstract has been revised, the changes are still insufficient. The Introduction should instead clearly describe the environmental and biogeochemical characteristics of the study area and position nitrogen fixation within that regional context. The title should also be revised accordingly.

The authors suggest that nitrogen fixation in the Barents Sea could serve as an important source of nitrogen. They also indicate in their rebuttal letter that the Barents Sea becomes oligotrophic in summer. However, apparent oligotrophy can also occur when newly supplied nutrients are rapidly consumed. A more direct way to evaluate the importance of nitrogen fixation would be to compare the measured nitrogen fixation rates with nitrate supply rates (Capone et al., 2005, GBC). Such a comparison would help determine whether nitrogen fixation represented a significant nitrogen source during the study period.

I am also concerned by the lack of presentation of environmental data. To interpret the microbial ecology of particle-associated nitrogen fixation, it is essential to describe the environmental conditions under which this activity was observed.

Minor comment:
The authors write that "A preliminary study estimated N2 fixation supports 17% of primary productivity in the region" (L58-59). Is this really correct? I checked the relevant literature, but I could not find such a description. A value corresponding to 17% of primary production is extraordinarily high. I think such a value would be rare even in low-latitude regions.

Reviewer #2

(Remarks to the Author)
I am satisfied with the author edits based on the reviews, thank you.

Open Access This Peer Review File is licensed under a Creative Commons Attribution 4.0 International License, which permits use, sharing, adaptation, distribution and reproduction in any medium or format, as long as you give appropriate credit to the original author(s) and the source, provide a link to the Creative Commons license, and indicate if changes were made.
In cases where reviewers are anonymous, credit should be given to 'Anonymous Referee' and the source.
The images or other third party material in this Peer Review File are included in the article's Creative Commons license, unless indicated otherwise in a credit line to the material. If material is not included in the article's Creative Commons license and your intended use is not permitted by statutory regulation or exceeds the permitted use, you will need to obtain permission directly from the copyright holder.
To view a copy of this license, visit https://creativecommons.org/licenses/by/4.0/

Response letter on reviews for manuscript

Please find below reviewers' comments in blue and our responses in black.

Reviewer #1 (Remarks to the Author):

Benavides et al shows that particle-associated microbes performed nitrogen fixation in the Atlantic Arctic, and those diazotrophs are non-cyanobacterial. I agree that this result is in itself a novel finding. However, I could not understand how impactful this discovery is.
Recent studies have already shown that particle-associated microbes perform nitrogen fixation in the ocean (Harding et al., 2022). Therefore, I am not particularly surprised that similar lifestyle diazotrophs also exist in the Arctic. For this reason, simply demonstrating the occurrence of nitrogen fixation by particle-associated diazotrophs may not be sufficient.

We thank the reviewer for raising these concerns. While particle-associated N_2 fixation is not a novel phenomenon, its occurrence in the Arctic Ocean represents, to the best of our knowledge, an important new finding considering this process has historically been considered to be restricted to (sub)tropical regions. We believe that the observation of particle-associated N_2 fixation rates in this environment being comparable to those in low-latitude waters is an important result to share with the scientific community, particularly given the expectation of substantially lower rates in colder environments. This is especially pertinent as the Arctic is becoming increasingly nitrogen-limited, potentially expanding the ecological niche for diazotrophs in the region.

Another point of concern is that the observation is limited to the Atlantic Arctic and is based on only a single cruise. How is the representativeness of this observation ensured? Based only on the title and abstract, readers may be left with the impression that this phenomenon occurs throughout the Arctic Ocean, but is that really justified? Environmental conditions differ substantially between the Atlantic and Pacific sectors of the Arctic because of differences in water mass inflow, and ecosystem responses also differ between these regions (Ardyna and Arrigo, 2020; Waite et al., 2026). I am not convinced that the authors fully appreciate this point. The Barents Sea, for example, is a region where mixing has intensified owing to the inflow of Atlantic Water, and primary production has correspondingly increased (Ingvaldsen et al., 2021: Noh et al., 2024). Because this production is driven mainly by nitrate supply, I question whether nitrogen fixation would in fact be an important process in such a region.

This is a fair critic, as the Atlantic and Pacific sectors of the Arctic are remarkably different physically and biogeochemically as the reviewer indicates. In terms of diazotrophy, reports have shown that the Atlantic sector is dominated by NCDs while the Pacific sector is more dominated by the *Braarudosphaera bigelowii* nitroplast (formerly UCYN-A) (Harding et al. 2018, von Friesen et al. 2025a b, Zander et al. 2025). However, we note that previous bulk and single-cell N_2 fixation measurements in the Arctic Pacific sector are comparable to the ones presented in our manuscript (Sipler et al. 2017, Harding et al. 2018).

We also agree that the Barents Sea is unique within the Arctic context, which may imply that the potential for N_2 fixation there differs from that in other Arctic regions. The Barents Sea is among the fastest warming regions of the Arctic. Here, increased primary productivity (Fig. 2D in (Ardyna & Arrigo 2020)) leads to nitrate depletion from May/June (Oziel et al. 2017, Henley et al. 2020), with low concentrations persisting throughout the summer (Henley et al. 2020). Tuerena et al. (Tuerena et al. 2022) further showed that, although weaker stratification and enhanced mixing may increase nutrient supply to surface waters in the Barents Sea, elevated primary productivity ultimately leads to a net depletion of upper-ocean nitrate during the summer. Together, these findings point to the Barents Sea as an important niche for N_2 fixation.

We modified the abstract, referred to the Atlantic Arctic sector in the introduction, and reviewed the particularities of the Barents Sea circulation, nitrate flux and limitations in being representative of N_2 fixation in the wider Arctic Ocean context in the Results and Discussion section lines 208-215. However, since to the best of our knowledge, no other study has measured particle-associated N_2 fixation in the Arctic, we respectfully believe the novelty justifies the manuscript title.

References

Ardyna, M., Arrigo, K.R. (2020) Phytoplankton dynamics in a changing Arctic Ocean, Nature Climate Change 10, 892-903.
Waite, A.M. et al. (2026) A quantitative comparison of the physical supply and biological uptake of new nitrogen in the Arctic Ocean, Nature reviews earth & environment, https://doi.org/10.1038/s43017-026-00769-z
Ingvaldsen, R.B. et al. (2021) Physical manifestations and ecological implications of Arctic Atlantification,

Nature reviews earth & environment 2, 874-889.
Noh, K. et al. (2024) Role of Atlantification in enhanced primary productivity in the Barents Sea, Earth's Future 21, e2023EF003709.

Reviewer #2 (Remarks to the Author):

This brief paper reports bulk nitrogen fixation rates from size fractionated samples collected from 14 stations in the Arctic subsurface (10 m below the chlorophyll max). A subset with the highest bulk rates were then analyzed for single cell N2 fixation. Solid paper, needs a few edits (see comments below)

Comments:

1) I found the use of the acronyms (susp, ss, fs, AW, wAW, ArW) in such a short paper to be tedious (it requires the reader to use their memory trying to remember what the acronyms are as they are trying to read and understand the paper). My suggestion is to avoid the acronyms as much as possible.

Agreed. We have written the abovementioned acronyms in full throughout the text but kept them in figures for simplicity (written in full in figure captions).

2) It does not appear that the authors used formalin-killed controls to determine the background isotope signal. It would be nice if the authors could state why they feel they do not require this, and if so, how they set up the nanoSIMS detectors to make sure the isotope ratios are correct. For example, do they run a natural abundance control sample first prior to running the isotope labeled samples?

We used filter areas without enrichment as background. This is explained in the Methods section lines 351-355: *"Additional ROIs were defined in areas without visible 15N enrichment to estimate internal background ratios, which were centred around natural abundance (~0.00367; ^{15}N atom %). Cells were considered isotopically enriched only when their 15N/14N ratios exceeded both natural abundance and the Poisson counting error associated with ion counting statistics."*

We used lab internal natural abundance samples for the adjustment of the detectors prior to running the enriched samples of the project and it was verified that the natural abundance isotope ratios are reached in Real Time Counting. This information has been updated in the Methods section lines 344-347.

3) Please discuss the size of the particles that were analyzed by nanoSIMS and convince the reader that topography was not an issue that could have resulted in incorrect isotope analyses (nanoSIMS works best on flat samples).

Particle size was estimated from image analysis by measuring the major and minor axes. Particles ranged from 14 to 182 µm in width (average 68 ± 41 µm). Particles were gently collected on polycarbonate filters using mild vacuum filtration. While care was taken to avoid structural damage, this process likely resulted in particles being flattened against the filter surface due to the flow, thereby reducing topographic complexity. In addition, microscopic observations did not reveal any regions out of focus within individual particles at the scale of observation, suggesting limited vertical relief and supporting the assumption that particles were relatively flat.

4) There is no discussion about the immunolabeling causing isotope dilution, therefore the N2 fixation rates reported are likely underestimates. Please address this somewhere.

We thank the reviewer for this point. Our procedure (NifH antibody staining) could certainly lead to a dilution of the 15N cell enrichment. PFA fixation would not because this fixative does not contain nitrogen. Contrary to Musat et al. (Musat et al. 2014) we do not run nanoSIMS analyses on samples after each step, so we cannot assess how they may have affected ^{15}N enrichment individually. Musat et al. also showed that the degree of isotopic dilution differs with growth phases, using cultured strains of *Pseudomonas*. We note that in our fieldwork setting, we cannot unfortunately assess the growth phase of the mixed microbial community sampled.

Nevertheless, considering the nitrogen content of the different chemicals used for antibody staining and considering the ^{15}N introduced into our sample (quantified by MIMS), we estimated the potential dilution of the signal. Sample were incubated in a blocking solution containing 5% (w/v) BSA (50 mg ml^{-1} of protein) and exposed to primary and secondary antibodies (1.2 µl and 1.1 µl in 200 µl respectively), which represent a negligible contribution compared to BSA. We assume that all these compounds contain nitrogen at natural isotopic balance (0.0366% ^{15}N). Only a small fraction of this bulk protein can interact with cells. Based on reported adsorption rates (0.05-1 µg cm^{-2}, up to 2.5 µg cm-2 (Guha Thakurta & Subramanian 2011)) and a bacterial surface around 3 x 10^{-8} cm^{-2} (Table S3), this corresponds to 1.5 fg of adsorbed protein, or 0.24 fg N per cell (assuming 16% nitrogen content of proteins). Given that a representative cell contains 100 fmol N (Table S3), a highly conservative worst-case scenario, assuming complete incorporation and mixing of this external nitrogen, yield a maximum dilution of 15%. This estimate in intentionally conservative, as it

neglects washing steps and assumes full intracellular incorporation of surface-bound proteins, which is unrealistic since proteins mainly remain adsorbed at the cell surface and are largely removed during washing. In practice, adsorption is largely restricted to the cell surface, and the actual dilution is expected to be substantially lower. This upped bound estimate is consistent with the dilution values reported by Musat et al. for CARD-FISH applications. We have included this information in the Supplementary Information text and referred to it in the Results and Discussion section.

5) Line 139: could you also have used chlorophyll or phycocyanin autofluorescence to determine if those cells were cyanobacteria?

Chlorophyll autofluorescence was not assessed because images were acquired in black and white using filter sets optimized for DAPI and Alexa Fluor 594, which do not allow detection of pigment autofluorescence. Retrospective identification is therefore, unfortunately, not possible. However, we would like to emphasize that the *nifH* amplicon data clearly shows that the particle-associated diazotroph community was almost exclusively composed of NCDs (Table S4, Fig. 3), and the small cell size (<2 µm for 85% of cells; Fig. S4) further supports the absence of significant cyanobacterial contribution.

6) Lines 147, 154 mention ASVs, but the main methods have nothing about 16S rRNA gene amplicon sequencing, perhaps a brief statement in the methods that the authors did 16S sequencing and refer the reader to the supplementary.

We did not do 16S sequencing, only *nifH* sequencing, which is the most widely used biomarker gene for N_2 fixation. For clarity, we have moved the *"DNA sampling, extraction, nifH gene sequencing and bioinformatics"* methods (previously in the Supplementary Information) to the main text Methods section.

7) Reading the main paper, I thought that nitrogen fixation was not measured in the free-living fraction (and it seemed to be ignored). But then I saw figure S3. It would be helpful to call this out specifically in the main paper. Also, how are there such high levels of free-living N2 fixers in the wAW fs samples? Aren't those supposed to be mostly particles? This needs a bit of an explanation.

We apologise for this confusion. We believe the reviewer refers to Fig. S2, that contains bulk N_2 fixation data. Bulk N_2 fixation rates were measured on non-size fractionated ('bulk') samples filtered onto GFF filters (0.7 µm nominal pore size) taken from the suspended, slow sinking and fast sinking compartments of the MSC (Fig. S2).

Subsamples of each MSC fraction were further filtered through 3 and 0.2 µm polycarbonate filters, representing the particle-associated and free-living fractions, respectively. Because we wanted to focus on particle-associated N_2 fixation, only 3 µm filters were analysed by nanoSIMS (and free-living diazotrophs were generally less abundant than particle-associated ones according to our immunofluorescence analyses, Fig. S3). We have clarified these aspects in the Results & Discussion and Methods sections, as well as modified the caption of Fig. 2 to specify that only particle-associated (3 µm filters) are included in the figure.

8) Fig 2B: the scale car of the isotopes is too small to see, and it would be helpful for the reader to have the fluorescence and isotope images be at the same scale.

We thank the reviewer for this suggestion. We have increased the size of the scale bar in the isotope images in Fig. 2B to improve visibility. Fluorescence and nanoSIMS images cannot unfortunately be shown at identical scale due to technical limitations of the workflow. Fluorescence imaging for particle relocation prior to nanoSIMS must be performed without immersion, and the Olympus BX61 used for this step is limited to a maximum dry objective of 20×. Consequently, fluorescence images cannot be acquired at the same magnification as nanoSIMS images.

9) Fig 3: why is it necessary to report the free-living microbial community identity if that fraction was not analyzed for N2 fixation? A short statement is enough.

We believe it is useful to document whether the particle-associated community is phylogenetically different from the free-living community. Generally, the diazotroph community composition in the Arctic has only been documented in a handful of studies, so we hope any new data would be useful to the community, even if not strictly necessary for the purpose of this study since we do not have accompanying free-living fraction nanoSIMS data.

10) Fig. S2: most of the N2 fixation is in the suspended fraction. Is most of that activity in the particles or in the free-living fraction?

We only analysed 3 µm filters with nanoSIMS (i.e., the particle-associated fraction), because it was the goal of our study. However, our immunofluorescence analyses suggest that diazotrophs were generally less abundant in the free-living fraction (Fig. S3).

Reviewer #3 (Remarks to the Author):

Benavides and colleagues measured nitrogen fixation in the Arctic, focusing specifically on nitrogen fixation on particles. The study uses immunolabelling of the NifH enzyme combined with nanoSIMS to measure 15N isotope incorporation at the single-cell level. The authors compare particles from different water masses as well as particles with different sinking rates, including suspended, fast-sinking, and slow-sinking particles. The study finds particle-associated nitrogen fixation may be a significant source of nitrogen in the Arctic and contributes the observed nitrogen fixation to non-cyanobacteial diazotrophs.

Overall, the manuscript is well organized, and the study provides novel data that fills a necessary gap in our understanding of nitrogen fixation and diazotrophs in the Arctic. However, I believe the manuscript could be improved by providing additional context, such as bulk whole water N2 fixation rates and the subsequent estimations of how much particulate N2 fixation contributes to the whole water values. Also, there are single-cell nitrogen fixation rates that are extremely high, without explanation or context for these values, it is plausible that these values are errors associated with the method. Further calculation of assimilation percent, which is used to estimate newly synthesized biomass based on isotope incorporation, would shed light on the biomass turnover behind the high nitrogen fixation rates (Popa et al., 2007).

We thank the reviewer for these relevant comments. We answer below to the different aspects brought up:

Bulk N_2 fixation: Bulk N_2 fixation rates from each MSC compartment are presented in Fig. S3 and used as a guidance to select which samples to analyse by nanoSIMS. Because particles are heterogeneously distributed in the water column, estimating the contribution of particle-associated activity to overall N_2 fixation would require knowledge of particle abundance by volume of seawater. i.e., for a given particle size range, by multiplying the ratio of N_2 fixing particles to non-N_2 fixing particles to the number of particles of that size range per litre of seawater, would provide an estimate of particle-associated N_2 fixation per whole litre of seawater. Unfortunately, we could not record particle abundance by volume of seawater data during our cruise, which would require deployment of optical particle profilers. It is certainly in our plans to incorporate LISST casts (Laser In-Situ Scattering and Transmissometry) in our upcoming Arctic expedition to bridge this gap.

Extremely high N_2 fixation rates: We thank the reviewer for flagging them. They were indeed errors from a given sample at station N10. This has been corrected (please see our more detailed answer to this reviewer's question in this regard below).

Calculating percent assimilation as in (Popa et al. 2007): This calculation requires comparing time zero and time final samples. We did not take individual time-zero samples due to the limited MSC material available, prioritising having triplicates instead of taking volume for natural 15N/14N ratio control. However, we filter areas without enrichment as background. This is explained in the Methods section lines 351-355: *"Additional ROIs were defined in areas without visible 15N enrichment to estimate internal background ratios, which were centred around natural abundance (~0.00367; ^{15}N atom %). Cells were considered isotopically enriched only when their 15N/14N ratios exceeded both natural abundance and the Poisson counting error associated with ion counting statistics."* We also used lab internal natural abundance samples for the adjustment of the detectors prior to running the enriched samples of the project and it verified that the natural abundance isotope ratios are reached in Real Time Counting, ensuring the quality of our nanoSIMS data (updated in lines 344-347).

Below are some additional comments and questions regarding the text.

Line 46: nitrogen, not nutrient

Corrected.

Line 117: is this percentage of the whole microbial community or the community present on particles?

Of the "total" community (free-living and particle-associated). This has been clarified in line 121.

Line 118: wAW waters, waters seems redundant based on the abbreviation

Corrected.

Line 124: how is 1192 fmol N cell-1 day-1 possible? That may be over 100% enrichment and considering the immunolabeling would dilute the isotope, it would in theory be even higher. Hypotheses for these values would be appreciated here or reference to discussing them later in the text.

We thank the reviewer for pointing this out. We checked our data and found that this and another four datapoints with rates ~500-1192 fmol N cell^{-1} d^{-1} corresponded to a single sample from station N10 to which the background had not been removed. These have been corrected in the original nanoSIMS image data pipeline and updated in Table S3, and in the text accordingly.

Line 127: In all water masses, the fs particles were higher than the susp particles? In line 100 the author says susp fraction had the highest N fix rates. Please address for clarity here.

We apologise for the confusion. The reference to higher N_2 fixation rates in the suspended fraction refers to bulk (and not single-cell) rates, lines 98-101: *"Bulk measurements across individual MSC fractions revealed that the suspended fraction had the highest N_2 fixation rates (up to 1.62 nmol N l^{-1} d^{-1}), followed by the fast sinking fraction (up to 0.46 nmol N l^{-1} d^{-1}), and the slow sinking fraction (up to 0.22 nmol N l^{-1} d^{-1}) (Fig. S2, Table S2)".*

Bulk N_2 fixation rates were measured on non-size fractionated ('bulk') samples filtered onto GFF filters (0.7 µm nominal pore size) taken from the suspended, slow sinking and fast sinking compartments of the MSC (Fig. S2). Subsamples of each MSC fraction were further filtered through 3 and 0.2 µm polycarbonate filters, representing the particle-associated and free-living fractions, respectively. Because we wanted to focus on particle-associated N_2 fixation, only 3 µm filters were analysed by nanoSIMS (and free-living diazotrophs were generally less abundant than particle-associated ones according to our immunofluorescence analyses, Fig. S3). We have clarified these aspects in the Results & Discussion and Methods sections, as well as modified the caption of Fig. 2 to specify that only particle-associated (3 µm filters) are included in the figure.

Line 148-152: Can the presence of chlorophyll by epifluorescence be used to verify if cyanobacteria were present? If not, why?

Chlorophyll autofluorescence was not assessed because images were acquired in black and white using filter sets optimized for DAPI and Alexa Fluor 594, which do not allow detection of pigment autofluorescence. Retrospective identification is therefore, unfortunately, not possible. However, we would like to emphasize that the *nifH* amplicon data clearly shows that the particle-associated diazotroph community was almost exclusively composed of NCDs (Table S4, Fig. 3), and the small cell size (<2 µm for 85% of cells; Fig. S4) further supports the absence of significant cyanobacterial contribution.

Line 154: top ten nifH AVSs. Also, was sequencing done on whole water samples or particle fractions and free-living fractions? So top 10 ASVs in what? A quick overview of the sequencing strategy here would help set up this paragraph from understanding.

The samples were size-fractionated prior to DNA extraction into particle-associated (>3 µm) and free-living (0.2-3 µm) fractions. The top 10 ASVs refer to the most abundant ASVs identified across the combined dataset (i.e. both fractions and all samples). Their relative abundances are then displayed separately for each fraction and water mass in Fig. 3. For clarity, the DNA extraction and sequencing methods have now been moved from the Supplementary Information to the main text under the Methods section.

Line 158: again what fraction was Desulfuro abundant?

In the particle-associated fraction of the suspended and fast-sinking compartments. This has been now clarified in line 170.

Line 168: I don't see the connection of the ice-laden sediment transport to the above reference to terrestrial origins, please expand your explanation.

We apologise for this unclearness. We have clarified the link between terrestrial/glacial inputs and ice-mediated transport of microbial communities, noting that ice and associated sediments can act as vectors carrying microorganisms from terrestrial and ice environments into offshore Arctic waters, thereby explaining the presence of typically soil- or ice-associated taxa in our pelagic samples. Lines 177-180: *"This suggests that the samples collected included particles of terrestrial and/or glacial origin, consistent with a recent study [21], and with the well-known role of sea ice and ice-rafted sediments in transporting microbial communities from terrestrial and cryospheric environments into open Arctic waters [48]."*

Line 177: The argument in this line is unclear

We agree this sentence was unclear. We have clarified as follows in lines 188-192: *"Alternatively, the scarcity of typical particle-associated pelagic diazotroph communities in our data may reflect the absence of phytoplankton-derived particles at our sampling depth (10 m below the deep chlorophyll maximum, 22-66*

m; Table S1, Fig. S1) due to remineralisation, consistent with particulate organic carbon attenuation horizons as shallow as 30-60 m in the Barents Sea [55]".

Line 184: tparticle, remove t

Corrected.

Line 186: Are the sequencing data from the Pacific and Atlantic part of this study or others?

The Pacific (Reeder et al. 2025) and Atlantic (Cerdán-García et al. 2024) sequencing data are part of other published studies from our group, bioproject numbers PRJNA108523 and PRJNA1 039751, respectively. These studies were conducted in the same way as the Arctic study presented here, allowing inter-comparison. Text in the supplementary methods explains how the different datasets were pooled and normalised for comparison.

Line 221: Did the 48-hour incubation cause more of the susp fraction to settle? Also, in all the fractions, it seems like settling over 48-hour incubations would create a larger space of particulate matter and potentially more anoxic zones that would not necessarily reflect the environment of sinking particle and would therefore affect the Nfix rates, potentially increasing the rates due to the incubation conditions.

We appreciate the reviewer pointing this out. We doubt suspended particles would settle in 48 h, as the suspended fraction captured by MSCs represents a persistent, dynamically maintained pool of marine snow that is not directly linked to export flux (Lampitt et al. 2000, Riley et al. 2012, Baumas et al. 2021). The particles collected in this study were small (average 68 ± 41 µm), likely resulting in low sinking velocities as previously observed in this region (Rogge et al. 2023).

Line 300: If Chlorophyll fluorescence was present, can it be used to identify cyanobacterial versus NCD cells

Kindly please refer to our response to your comment on "Line 148-152" above.

Line 569: The presence of NifH and DAPI does not necessarily indicate active N2 fixation; the incorporation of 15N does, however.

Agreed. The presence of NifH protein and DAPI staining indicates the presence of diazotrophs with the potential for N_2 fixation, while 15N incorporation provides direct evidence of active N_2 fixation. We have updated the caption of Figure 2 accordingly.

References

Popa, R. et al. Carbon and nitrogen fixation and metabolite exchange in and between individual cells of Anabaena oscillarioides. ISME J 1,354–360 (2007).

References used in responses to reviewers

Ardyna M, Arrigo KR (2020) Phytoplankton dynamics in a changing Arctic Ocean. Nat Clim Chang 10:892–903.

Baumas CMJ, Le Moigne FAC, Garel M, Bhairy N, Guasco S, Riou V, Armougom F, Grossart H-P, Tamburini C (2021) Mesopelagic microbial carbon production correlates with diversity across different marine particle fractions. ISME J.

Cerdán-García E, Álvarez-Salgado XA, Arístegui J, Martínez-Marrero A, Benavides M (2024) Eddy-driven diazotroph distribution in the subtropical North Atlantic: horizontal variability prevails over particle sinking speed. Commun Biol 7:929.

von Friesen LW, Laber CP, Kristensen BH, Nysted E, Sundbom M, Bertilsson S, Snoeijs-Leijonmalm P, Farnelid H, Riemann L (2025a) From temperate to polar waters: Transition to non-cyanobacterial diazotrophy upon entering the Atlantic gateway of the Arctic Ocean. Limnology and Oceanography.

von Friesen LW, Löscher CR, Bertilsson S, Farnelid H, Snoeijs-Leijonmalm P, Sundbom M, Traving SJ, Vermassen F, Riemann L (2025b) Nitrogen fixation potential in bathypelagic sediment of the ice-covered Arctic Ocean revealed through long-term stable isotope incubations. Environ Microbiol Rep 17:e70173.

von Friesen LW, Paulsen ML, Müller O, Gründger F, Riemann L (2023) Glacial meltwater and seasonality influence community composition of diazotrophs in Arctic coastal and open waters. FEMS Microbiol Ecol 99.

Guha Thakurta S, Subramanian A (2011) Evaluation of in situ albumin binding surfaces: a study of protein adsorption and platelet adhesion. J Mater Sci Mater Med 22:137–149.

Harding K, Turk-Kubo KA, Sipler RE, Mills MM, Bronk DA, Zehr JP (2018) Symbiotic unicellular cyanobacteria fix nitrogen in the Arctic Ocean. Proceedings of the National Academy of Sciences:201813658.

Henley SF, Porter M, Hobbs L, Braun J, Guillaume-Castel R, Venables EJ, Dumont E, Cottier F (2020) Nitrate supply and uptake in the Atlantic Arctic sea ice zone: seasonal cycle, mechanisms and drivers. Philos Trans A Math Phys Eng Sci 378:20190361.

Krumpen T, Belter HJ, Boetius A, Damm E, Haas C, Hendricks S, Nicolaus M, Nöthig E-M, Paul S, Peeken I, Ricker R, Stein R (2019) Arctic warming interrupts the Transpolar Drift and affects long-range transport of sea ice and ice-rafted matter. Sci Rep 9:5459.

Lampitt RS, Newton PP, Jickells TD, Thomson J, King P (2000) Near-bottom particle flux in the abyssal northeast Atlantic. Deep Sea Res Part 2 Top Stud Oceanogr 47:2051–2071.

Musat N, Stryhanyuk H, Bombach P, Adrian L, Audinot J-N, Richnow HH (2014) The effect of FISH and CARD-FISH on the isotopic composition of (13)C- and (15)N-labeled Pseudomonas putida cells measured by nanoSIMS. Syst Appl Microbiol 37:267–276.

Oziel L, Neukermans G, Ardyna M, Lancelot C, Tison J-L, Wassmann P, Sirven J, Ruiz-Pino D, Gascard J-C (2017) Role for Atlantic inflows and sea ice loss on shifting phytoplankton blooms in the Barents Sea: SHIFTING BLOOMS IN THE BARENTS SEA. J Geophys Res Oceans 122:5121–5139.

Popa R, Weber PK, Pett-Ridge J, Finzi JA, Fallon SJ, Hutcheon ID, Nealson KH, Capone DG (2007) Carbon and nitrogen fixation and metabolite exchange in and between individual cells of Anabaena oscillarioides. ISME J 1:354–360.

Reeder CF, Filella A, Voznyuk A, Coët A, James RC, Rohrer T, White AE, Berline L, Grosso O, van Dijken G, Arrigo KR, Mills MM, Turk-Kubo KA, Benavides M (2025) Unveiling the contribution of particle-associated non-cyanobacterial diazotrophs to N2 fixation in the upper mesopelagic North Pacific Gyre. Commun Biol 8:287.

Riley JS, Sanders R, Marsay C, Le Moigne FAC, Achterberg EP, Poulton AJ (2012) The relative contribution of fast and slow sinking particles to ocean carbon export. Global Biogeochem Cycles 26.

Rogge A, Janout M, Loginova N, Trudnowska E, Hörstmann C, Wekerle C, Oziel L, Schourup-Kristensen V, Ruiz-Castillo E, Schulz K, Povazhnyy VV, Iversen MH, Waite AM (2023) Carbon dioxide sink in the Arctic Ocean from cross-shelf transport of dense Barents Sea water. Nat Geosci 16:82–88.

Sipler RE, Gong D, Baer SE, Sanderson MP, Roberts QN, Mulholland MR, Bronk DA (2017) Preliminary estimates of the contribution of Arctic nitrogen fixation to the global nitrogen budget. Limnology and Oceanography Letters 2:159–166.

Tuerena RE, Mahaffey C, Henley SF, de la Vega C, Norman L, Brand T, Sanders T, Debyser M, Dähnke K, Braun J, März C (2022) Nutrient pathways and their susceptibility to past and future change in the Eurasian Arctic Ocean. Ambio 51:355–369.

Zander S, von Friesen LW, Gonçalves-Araujo R, Grosso O, Benavides M, Granskog MA, Riemann L (2025) Contrasting nitrogen fixation between arctic and Atlantic waters in the Fram Strait. Microb Ecol 89:26.

Response letter on reviews for manuscript

Please find below reviewers' comments in blue and our responses in black.

Reviewer #1 (Remarks to the Author):

My impression has not changed after reading their rebuttal letter and revised manuscript. It may indeed be true that this is the first study to observe nitrogen fixation in particles in the Arctic Ocean. However, the importance of Harding et al. (2022) lies in the fact that they were the first to directly measure nitrogen fixation activity in particles. Even before that study, it was already known that organisms with the potential for nitrogen fixation could occur in particles. The value of Harding et al. (2022) was that they demonstrated, for the first time, that nitrogen fixation activity actually occurs within particles.
If diazotrophs were predominantly particle-associated across the pan-Arctic Ocean, this would represent an interesting ecological pattern distinct from those observed in other oceanic regions. However, the available evidence does not support such a broad interpretation. In the Pacific sector of the Arctic Ocean, UCYN-A has been shown to actively contribute to nitrogen fixation (Harding et al., 2018), as the authors themselves seem to acknowledge in their rebuttal letter. Thus, the authors' observations are better interpreted as a local phenomenon occurring in part of the Arctic Ocean, rather than as a general feature of Arctic nitrogen fixation.
I do not see serious flaws in the authors' analytical methods. My main concern is that the authors appear to draw conclusions that go beyond what their results can support. This tendency is also evident in the framing of the Introduction.
A study conducted in the Barents Sea should not be framed as representing the pan Arctic Ocean. Although the Abstract has been revised, the changes are still insufficient. The Introduction should instead clearly describe the environmental and biogeochemical characteristics of the study area and position nitrogen fixation within that regional context. The title should also be revised accordingly.

We thank the reviewer for their comments. We have specified "Barents Sea" instead of "Arctic" (including the title). Additional considerations about the particularities of this region vs the wider Arctic context in the abstract, introduction, and results & discussion sections. We have added extra text on sampling across environmental gradients in the Supplementary Information (further details covered in a recent companion publications and datasets (Ilyas et al. 2026, Lohan et al. 2026, Mahaffey et al. 2026, Hawley et al. 2026).

The authors suggest that nitrogen fixation in the Barents Sea could serve as an important source of nitrogen. They also indicate in their rebuttal letter that the Barents Sea becomes oligotrophic in summer. However, apparent oligotrophy can also occur when newly supplied nutrients are rapidly consumed. A more direct way to evaluate the importance of nitrogen fixation would be to compare the measured nitrogen fixation rates with nitrate supply rates (Capone et al., 2005, GBC). Such a comparison would help determine whether nitrogen fixation represented a significant nitrogen source during the study period.

We agree with this reviewer that a direct comparison with nitrate fluxes would help place our N_2 fixation rates in context. However, as noted in our previous response to another reviewer (pasted below), the limitation lies in the nature of the rates being measured:

"Because particles are heterogeneously distributed in the water column, estimating the contribution of particle-associated activity to overall N2 fixation would require knowledge of particle abundance by volume of seawater. i.e., for a given particle size range, by multiplying the ratio of N2 fixing particles to non-N2 fixing particles to the number of particles of that size range per litre of seawater, would provide an estimate of particle-associated N2 fixation per whole litre of seawater. Unfortunately, we could not record particle abundance by volume of seawater data during our cruise, which would require deployment of optical particle profilers. It is certainly in our plans to incorporate LISST casts (Laser In-Situ Scattering and Transmissometry) in our upcoming Arctic expedition to bridge this gap".

Our rates are measured in individual cells attached to individual particles with units fmol N $cell^{-1}$ d^{-1}. Comparing particle-associated N_2 fixation rates with nitrate fluxes would require volumetric rates (i.e. nmol N l^{-1} d^{-1}) or areal rates if integrated in the water column (i.e. mmol m^{-2} d^{-1}). Extrapolating particle-associated rates to volumetric or areal rates requires (a) knowledge of the concentration of particles per volume of seawater, and (b) more than one measurement in the water column in order to be able to integrate values vertically in the water column. We unfortunately do not have either a or b, which precludes us from comparing particle-associated N_2 fixation rates to nitrate flux rates. Any attempt to make this calculation with the data in hand would be fraught with caveats and lead to an estimate in the literature that is poorly constrained.

This was written in the previously submitted version of this manuscript as follows:
"Future assessments of the contribution of particle-associated activity to overall N_2 fixation will require detailed knowledge of particle size spectra and abundance in the water column, obtained using optical methods such as Laser In-Situ Scattering and Transmissometry."

We have attempted a back-of-the-envelope calculation to place our results in context. Assuming an average abundance of 4×10^4 particles l^{-1} within our size range (Fig. S8), based on measurements from the Barents Sea (Lokhov et al. 2020) together with our particle-associated single-cell N_2 fixation rates (Table S3), an average of two N_2-fixing cells per particle, and a homogeneous distribution throughout the upper 50 m of the water column, we estimate water-column-integrated particle-associated N_2 fixation rates ranging from 0 to 1.61 mmol N m^{-2} d^{-1} (average 0.02 mmol N m^{-2} d^{-1}). Compared with the average nitrate flux reported for the Barents Sea by (Waite et al. 2026) of 4.5 mmol N m^{-2} d^{-1} particle-associated N_2 fixation could therefore contribute between 0 and 35.8% of the nitrogen supply in this region.

However, these estimates rely on several highly uncertain assumptions, including particle abundance, the number of diazotrophs per particle, their spatial distribution, and the extrapolation of single-cell rates to the water-column scale. The resulting contribution spans nearly two orders of magnitude, highlighting the substantial uncertainty associated with this exercise. We therefore feel that including these values in the manuscript could be misleading, and we are conscious of putting such poorly constrained estimates in the literature to be used by others. Instead, we believe it is more appropriate to reserve such quantitative comparisons for future studies that combine direct measurements of particle-associated N_2 fixation with particle abundance profiles (e.g., LISST observations), allowing for a more robust assessment of the contribution of particle-associated N_2 fixation to the nitrogen budget of the Barents Sea.

Nevertheless, we would like to stress that the clear predominance of particle-associated diazotrophs in the Barents Sea as compared to the two subtropical reference sites suggests particle-associated N_2 fixation is relevant in this region. As this reviewer states, this may not be the case elsewhere in the Arctic, so we have modified the text to make clear our interpretations refer exclusively to the Barents Sea.

I am also concerned by the lack of presentation of environmental data. To interpret the microbial ecology of particle-associated nitrogen fixation, it is essential to describe the environmental conditions under which this activity was observed.

The environmental conditions are described in a recently published companion paper (Hawley et al. 2026) and the dominant water masses present at each station can be found in (Ilyas et al. 2026) which are also used in (Hawley et al. 2026). The nutrients, trace metal, oxygen, etc, and CTD profiles corresponding to our MSC deployments are included in our manuscripts Supplementary Information.

To place more of this hydrographic context into the main body of the manuscript we have included a short paragraph in the Supplementary Information that describes the overall south to north transition from Atlantic to Arctic dominated waters, corresponding latitudinal trends in the depth of the seasonal surface mixed layer depth and subsurface chlorophyll-maximum.

Minor comment:
The authors write that "A preliminary study estimated N2 fixation supports 17% of primary productivity in the region" (L58-59). Is this really correct? I checked the relevant literature, but I could not find such a description. A value corresponding to 17% of primary production is extraordinarily high. I think such a value would be rare even in low-latitude regions.

We thank the reviewer for spotting this error. The (Sipler et al. 2017) paper reports maximum N_2 fixation rates of 17.2 nmol N l^{-1} d^{-1} or 9.2 Tg N y^{-1}, not of percentage contribution to primary productivity. It has been corrected in the text.

References

Hawley R, Mahaffey C, Hopkins J, Norman L, Flanagan O, Rynders S, Barton BI, Sanders RNC, Meredith MP, Arrowsmith C, Lohan MC (2026) Iron and manganese cycling in the atlantifying Barents Sea: Concentrated inputs and emerging limitations. Global Biogeochem Cycles 40:e2025GB009031.

Ilyas M, Hopkins J, Mahaffey C, Norman L (2026) Classification of Barents Sea water masses from cruise DY167 (RRS Discovery) in July-August 2023 using Optimum Multiparameter (OMP) analysis.
Lohan M, Hawley R, Flanagan O, Mahaffey C, Norman L, Hopkins J, Barton BI, Henderson P, Rynders S (2026) Titanium CTD water column samples (macronutrients, chlorophyll-a, trace elements) and upcast sensor measurements (temperature, salinity, oxygen, optical backscatter, beam transmittance and attenuation, PAR, fluorescence) from the Norwegian and Barents Sea on cruise DY167 (GApr19), July-August 2023.
Lokhov AS, Kravchishina MD, Klyuvitkin AA, Kochenkova AI (2020) In situ measurements of the characteristics of suspended particles in the Barents sea by the LISST-deep laser diffractometer. Russ Acad Sci Oceanol 60:650–663.
Mahaffey C, Fisher BJ, Wrightson L, Hopkins J, Arrowsmith C, Sanders RN, Meredith MP, Barton BI, Henderson P, Rynders S (2026) Stainless Steel CTD water column samples (macronutrients, chlorophyll-a, particulate carbon, C-fixation, d18O) and upcast sensor measurements (temperature, salinity, oxygen, optical backscatter, beam transmittance and attenuation, PAR, fluorescence) from cruise DY167 (GApr19), July-August 2023.
Sipler RE, Gong D, Baer SE, Sanderson MP, Roberts QN, Mulholland MR, Bronk DA (2017) Preliminary estimates of the contribution of Arctic nitrogen fixation to the global nitrogen budget. Limnology and Oceanography Letters 2:159–166.
Waite AM, Lane A, Carmack E, Danielson SL, Ellingsen I, Fer I, Lenn Y-D, Oziel L, Rabe B, Randelhoff A, Raes EJ, Rogge A, Tremblay J-É, Torres-Valdés S, Weil S-S, von Appen W-J (2026) A quantitative comparison of the physical supply and biological uptake of new nitrogen in the Arctic Ocean. Nat Rev Earth Environ:1–18.
