# Supplementary material for: Particle-associated diazotrophs drive nitrogen fixation in Arctic subsurface waters of the Barents Sea
Source: Nat Commun. 2026 Jul 8;17:8417. doi: 10.1038/s41467-026-75354-5 (PMC13478305; doi:10.1038/s41467-026-75354-5)
Supplement: Supplementary file 3 [file 41467_2026_75354_MOESM3_ESM.pdf]

Description of Additional Supplementary Files

File Name: Supplementary Data 1
Description: **Sampling station metadata.** List and classification of sampled stations and type of sampling conducted at each. Seawater mass classification was based on ref [16] .

File Name: Supplementary Data 2
Description: **Bulk N_2 fixation rates.** Bulk N2 fixation rates by marine snow catcher (MSC) fractions, station number, and water mass (AW = Atlantic Water, wAW = warm Atlantic Water, ArW = Arctic Water).

File Name: Supplementary Data 3
Description: **Particle-associated N_2 fixation rates.** ^{15}N atom % enrichment, cell diameter, biovolume, particulate carbon (PC), particulate nitrogen (PN) and N_2 fixation rates of single-cells measured from 3 µm filters on suspended (susp) and fast sinking (fs) marine snow catcher (MSC) fractions.

File Name: Supplementary Data 4
Description: **Amplicon sequence variants.** Taxonomic annotation of amplicon sequence variants (ASVs).

File Name: Supplementary Data 5
Description: **Metadata used in multivariate redundancy analysis.** Environmental metadata used in redundancy analysis (RDA, Fig. S5).
